# Characterization of long-chain fatty acid-linked bile acids: a major conjugation form of 3β-hydroxy bile acids in feces

**DOI:** 10.1016/j.jlr.2022.100275

**Published:** 2022-09-09

**Authors:** Hajime Takei, Seiko Narushima, Mitsuyoshi Suzuki, Genta Kakiyama, Takahiro Sasaki, Tsuyoshi Murai, Yuichiro Yamashiro, Hiroshi Nittono

**Affiliations:** 1 Junshin Clinic Bile Acid Institute, Meguro-ku, Tokyo, Japan; 2 RIKEN Center for Integrative Medical Sciences, Yokohama City, Kanagawa, Japan; 3 Department of Pediatrics, Juntendo University, Faculty of Medicine, Bunkyo-ku, Tokyo, Japan; 4 Division of Gastroenterology, Hepatology, and Nutrition, Virginia Commonwealth University, School of Medicine and Central Virginia VA Healthcare System, Richmond, VA; 5 Faculty of Pharmaceutical Sciences, Health Sciences University of Hokkaido, Ishikari, Hokkaido, Japan; 6 Probiotics Research Laboratory, Juntendo University Graduate School of Medicine, Bunkyo-ku, Tokyo, Japan

**Keywords:** fecal bile acid, saponifiable bile acids, LC/MS, precursor ion scan, FA-conjugated isobile acids, BA, bile acid, CA, cholic acid, CDCA, chenodeoxycholic acid, CID, collision-induced dissociation, DCA, deoxycholic acid, FA-isoBA, Fatty acid-conjugated isobile acid, GCA, glycocholic acid, GCDCA, glycochenodeoxycholic acid, IS, internal standard, isoDCA, isodeoxycholic acid, isoLCA, isolithocholic acid, LCA, lithocholic acid, LCA-3S, lithocholic acid 3-sulfate, Lin, linoleic acid, LOQ, limit of quantification, Ole, oleic acid, Pam, palmitic acid, Ste, stearic acid, TBA, total bile acid, TCA, taurocholic acid, TCDCA, taurochenodeoxycholic acid, UDCA, ursodeoxycholic acid

## Abstract

Although most bile acids (BAs) in feces are present in noncovalent forms that can be extracted with ethanol, non-negligible amounts of saponifiable BAs are also present. It is a major concern that such saponifiable BAs are routinely omitted from fecal BA measurements. We compared the BA profiles of healthy stools that were obtained with/without alkaline hydrolysis and found that as much as 29.7% (2.1–67.7%) of total BAs were saponifiable. Specifically, alkaline treatment led to significant elevations of isodeoxycholic acid (isoDCA) and isolithocholic acid (isoLCA) concentrations, suggesting that considerable proportions of isoDCA and isoLCA were esterified. Precursor ion scan data from LC/MS suggested the presence of long-chain FA-linked BAs. We chemically synthesized a series of fatty acid 3β-acyl conjugates of isoDCA and isoLCA as analytical standards and analyzed their fecal profiles from newborns to adults (n = 64) by LC/MS. FA-conjugated isobile acids (FA-isoBAs) were constantly present from 2 years of age to adulthood. C16- and C18-chain FA-isoBA esters were predominantly found regardless of age, but small amounts of acetic acid esters were also found. FA-isoBA concentrations were not correlated to fecal FA concentrations. Interestingly, there were some adults who did not have FA-isoBAs. Gut bacteria involved in the production of FA-isoBAs have not been identified yet. The present study provides insight into the establishment of early gut microbiota and the interactive development of esterified BAs.The contribution of FA-isoBAs to gut physiology and their role in pathophysiologic conditions such as inflammatory bowel disease are currently under investigation.

Bile acids (BAs), the main catabolic products of cholesterol metabolism, play an essential role in the digestion and absorption of lipids from the diet (1, 2). They also serve as signaling molecules involved in various physiological processes, such as the regulation of lipid, glucose, and energy metabolism (3, 4). In the intestinal lumen, BAs represent distinctive molecules that tightly interplay with gut microbes and host physiology. As important messengers between the gut and the liver, BAs can influence lipid and energy metabolism, as well as impact immune responses (5, 6, 7). Epidemiological studies have shown that there is a clear correlation between events that disrupt the gut microbiota during childhood and the occurrence of immune and/or metabolic disorders later in life (8, 9, 10).

Primary conjugated BAs synthesized in the liver are secreted into the gastrointestinal tract with bile, reabsorbed in the intestine, and transported back to the liver. In humans, the enterohepatic circulation of BAs is very efficient. Most BAs (95%) are reabsorbed from the brush border membrane of the terminal ileum, and only a small portion (5%) reach the large intestine and are excreted into feces. During passage of the gastrointestinal tract, conjugated BAs are deconjugated and modified by gut bacteria with reactions such as dehydroxylation, oxidation, epimerization of hydroxyl groups, and esterification with other biomolecules. The deconjugation of primary BAs is mediated by bile salt hydrolase, which is present in many bacteria, including gram-negative *Bacteroides* and gram-positive *Lactobacillus* and *Clostridium*. Cholic acid (CA) and chenodeoxycholic acid (CDCA) can be 7α-dehydroxylated to form deoxycholic acid (DCA) and lithocholic acid (LCA), respectively (11). Only a small population of intestinal species in the genus *Clostridium*, including *Clostridium scindens* (Clostridium cluster XIVa), *Clostridium hiranonis* (Clostridium cluster XI), *Clostridium hylemonae* (Clostridium cluster XIVa), and *Clostridium sordellii* (Clostridium cluster XI) are capable of this biotransformation. In humans, *Clostridium* colonizes in the intestine soon after weaning (12, 13). Concomitantly, fecal DCA and LCA levels are significantly elevated as of 26–27 weeks of age (14). In addition, CDCA can be transformed into ursodeoxycholic acid (UDCA) by 7α-/7β-hydroxysteroid dehydrogenases via 7α/β-epimerization (15). These secondary BAs can also be passively absorbed from the gut and function as signaling molecules. Moreover, some types of BAs (e. g., isoLCA) can be esterified with long-chain fatty acids (FAs) (e.g., palmitic acid [Pam], palmitoleic acid, stearic acid [Ste], and oleic acid [Ole]) (16). Thus, more than 50 secondary bile acids and their conjugated forms have been found in human feces (17, 18).

Fecal BAs can reflect intestinal susceptibility to disease. There are a number of reports that have measured fecal BA profiles in healthy and diseased states such as colorectal cancer (19), liver cancer (20), ulcerative colitis (21), cirrhosis (22), and in centenarians (23). Although recent advances in LC/MS technology have dramatically improved BA profiling (24), the presence of many isomers as well as various types of conjugated forms still complicates the separation and detection of fecal BAs. In fact, esterified BAs are routinely omitted from the measurements. Commonly, ethanol or weak alkaline solutions have been used for the extraction of fecal BAs (25, 26, 27) without complete saponification of the esterified BAs. Thus, there are only a few reports that have found abundant saponifiable BAs in feces (16, 28, 29, 30). Previously, we analyzed fecal BAs with/without alkaline hydrolysis and reported that a considerable amount of saponifiable BA can be present in healthy and cirrhotic subjects (22).

To optimize fecal BA quantification, we re-examined the sample preparation procedure using various fecal samples obtained from infants to adults. Interestingly, when feces were refluxed in 1 M methanolic KOH for 1 h, total BA concentrations plateaued, and these concentrations were markedly higher than those obtained by the previous method with milder alkaline treatment (22). The concentrations of isoDCA and isoLCA were remarkably elevated compared to other BAs with the described treatment. MS precursor scan data suggested the presence of several kinds of long-chain FA-linked isoDCA and isoLCA. We chemically synthesized a series of FA-esterified isoDCA and isoLCA as analytical standards for LC/MS analysis. After validation of the method, we measured comprehensive fecal BA profiles in 64 healthy subjects from newborns to adults. Here, we report the quantification of FA-esterified BAs in human feces without saponification for the first time. The results provide an insight into the establishment of early gut microbiota and its interactive development of esterified BAs.

## Materials and methods

### Chemicals and reagents

All solvents and reagents except those described below were purchased from Kanto Chemical Co., Inc. (Tokyo, Japan). Analytical reagent-grade and LC/MS-grade solvents were used for the sample preparation and the LC/MS mobile phase, respectively. Dimethylazodicarboxylate, N,N′-diisopropylcarbodiimide, dimethylaminopyridine, and lithium iodide were purchased from FUJIFILM Wako Pure Chemical Corporation (Miyazaki, Japan). A Bond Elut C18 (500 mg/6 ml) solid phase extraction cartridge was obtained from Agilent (Tokyo, Japan).

Abbreviations and trivial names of BAs used in this study are shown in the supplemental Table S1. Standard products such as Pam, Ste, Ole, and linoleic acid (Lin) were purchased from FUJIFILM Wako Pure Chemical Corporation. *d*_3_-Pam and ^13^C_18_-Ste were purchased from Sigma Chemicals (St. Louis, MO). CA, CDCA, UDCA, DCA, LCA, and hyocholic acid were also purchased from Sigma Chemicals. [2,2,3,4,4-*d*_5_]-CA (*d*_5_-CA, internal standard (IS) for trihydroxy BAs), [2,2,4,4-*d*_4_]-DCA (*d*_4_-DCA, IS for dihydroxy BAs), [2,2,4,4-*d*_4_]-LCA (*d*_4_-LCA, IS for monohydroxy BAs), [2,2,4,4-*d*_4_]-glycocholic acid (GCA) (*d*_4_-GCA, IS for glycine-conjugated trihydroxy BAs), [2,2,4,4-*d*_4_]-taurocholic acid (TCA) (*d*_4_-TCA, IS for taurine-conjugated trihydroxy BAs), [2,2,4,4-*d*_4_]-glycochenodeoxycholic acid (GCDCA) (*d*_4_-GCDCA, IS for glycine-conjugated di- and mono-hydroxy BAs), and [2,2,4,4-*d*_4_]-taurochenodeoxycholic acid (TCDCA) (*d*_4_-TCDCA, IS for taurine-conjugated di- and mono-hydroxy BAs) were obtained from CDN Isotopes (Quebec, Canada). AlloCA, isoCA, 3-oxo-CA, 7-oxo-DCA, 12-oxo-CDCA, UCA, isoUCA, alloCDCA, isoCDCA, 3-oxo-CDCA, 3-oxo-alloCDCA, 7-oxo-LCA, UDCA, isoUDCA, 3-oxo-UDCA, alloDCA, isoDCA, isoalloDCA, 3-oxo-DCA, 3-oxo-alloDCA, 12β-OH-LCA, 12β-OH-isoLCA, 12-oxo-LCA, 12-oxo-isoLCA, alloLCA, isoLCA, isoalloLCA, 3-oxo-LCA, 3-oxo-alloLCA, hyodeoxycholic acid, murideoxycholic acid, CA-Δ^4^-3-one, DCA-Δ^4,6^-3-one, CDCA-Δ^4^-3-one, LCA-Δ^4,6^-3-one, DCA-Δ^4^-3-one, LCA-Δ^4^-3-one, nor-CA, GCA-Δ^4^-3-one, TCA-Δ^4^-3-one, GCDCA-Δ^4^-3-one, and TCDCA-Δ^4^-3-one were gifts from Professor Takashi Iida (Nihon University, Tokyo Japan). CA-3S, CDCA-3S, UDCA-3S, DCA-3S, lithocholic acid 3-sulfate (LCA-3S), GCA-3S, GCDCA-3S, GUDCA-3S, GDCA-3S, GLCA-3S, TCA-3S, TCDCA-3S, TUDCA-3S, TDCA-3S, TLCA-3S, [2,2,3,4,4-*d*_5_]-GCDCA-3S (*d*_5_-GCDCA-3S, IS for glycine-conjugated BA 3-sulfates), [2,2,3,4,4-*d*_5_]-TCDCA-3S (*d*_5_-TCDCA-3S, IS for taurine-conjugated BA 3-sulfates), and [2,2,3,4,4-*d*_5_]- CDCA-3S (*d*_5_-CDCA-3S, IS for BA 3-sulfates) were synthesized using previously reported methods (31).

### Chemical synthesis of FA-conjugated isoLCA and isoDCA for analytical standards

**Fig. 1:**
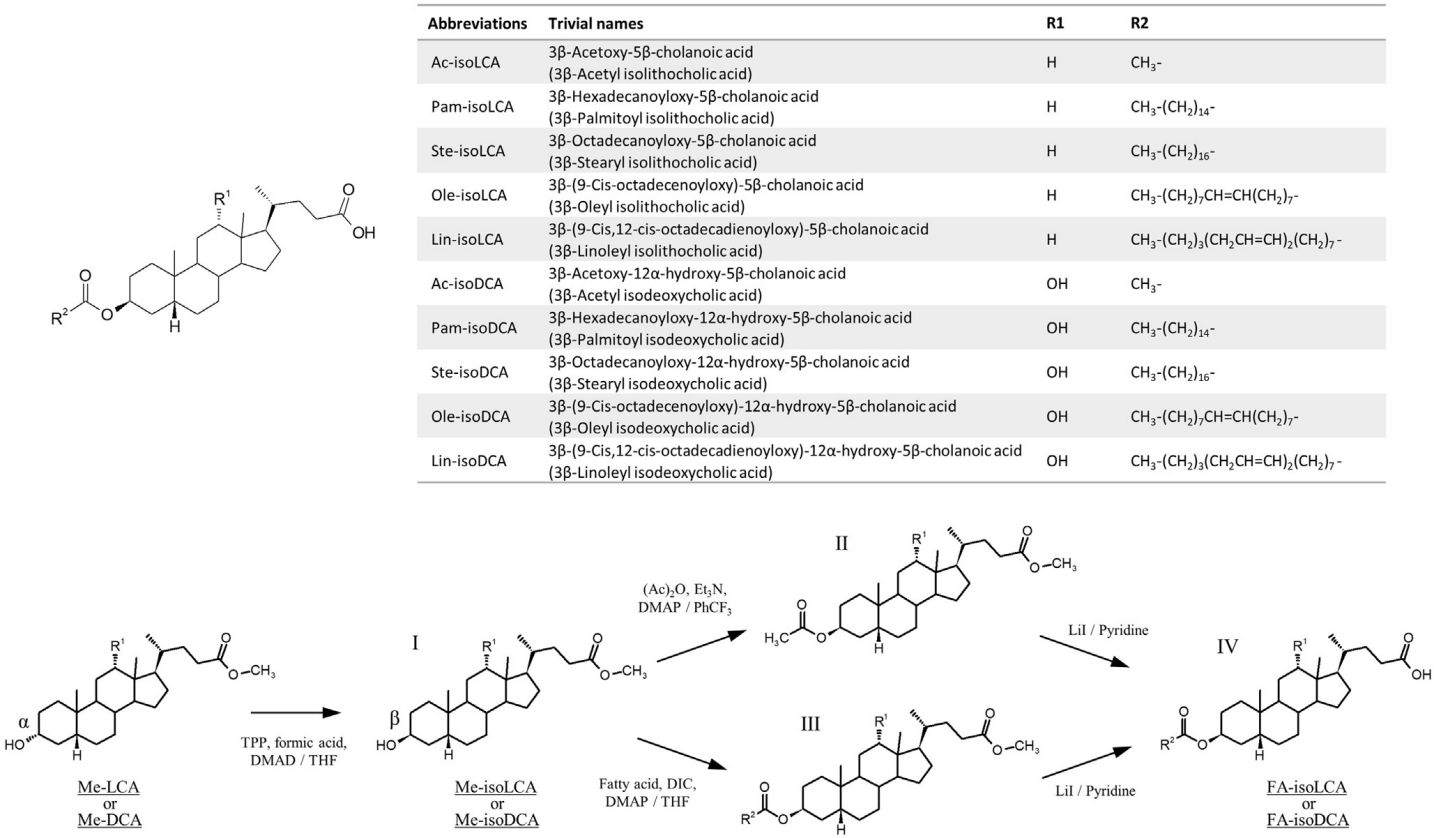
Chemical structure and synthesis of FA-BA esters. The detailed procedure as well as NMR spectral data are shown in Supplementary text S1. DMAD, dimethylazodicarboxylate; DIC, *N,N′*-diisopropylcarbodiimide; DMAP, dimethylaminopyridine; Et_3_N, triethylamine; LiI, lithium iodide; THF, tetrahydrofuran; TPP, triphenylphospine; PhCF_3_, benzotrifluoride; BA, bile acid; FA, fatty acid.

A series of FA 3β-acyl-conjugated BAs (FA-isoBAs) was chemically synthesized by the three-step procedure shown in Fig. 1. The detailed procedures and NMR spectral data of the synthetic FA-isoBAs are described in supplemental Text S1.

### Preparation of standard and IS solutions

Individual stock solutions of BAs, FAs, and FA-isoBAs in methanol (1 mg/ml) were prepared separately and stored at −20°C. BA standard mixtures were prepared at 10, 30, 100, 300, 1,000, 3,000, and 10,000 pmol/ml in 60% methanol; FA standard mixtures were prepared at 300, 1,000, 3,000, 10,000, 3,000, and 100,000 pmol/ml in 60% methanol; and FA-isoBAs standard mixtures were prepared at 1, 3, 10, 30, 100, 300, and 1,000 pmol/ml in 80% methanol. IS solutions were also prepared in methanol (100 nmol/ml for BAs, 1,000 nmol/ml for FAs, and 10 nmol/ml for FA-isoBAs).

### Sample processing

**Fig. 2:**
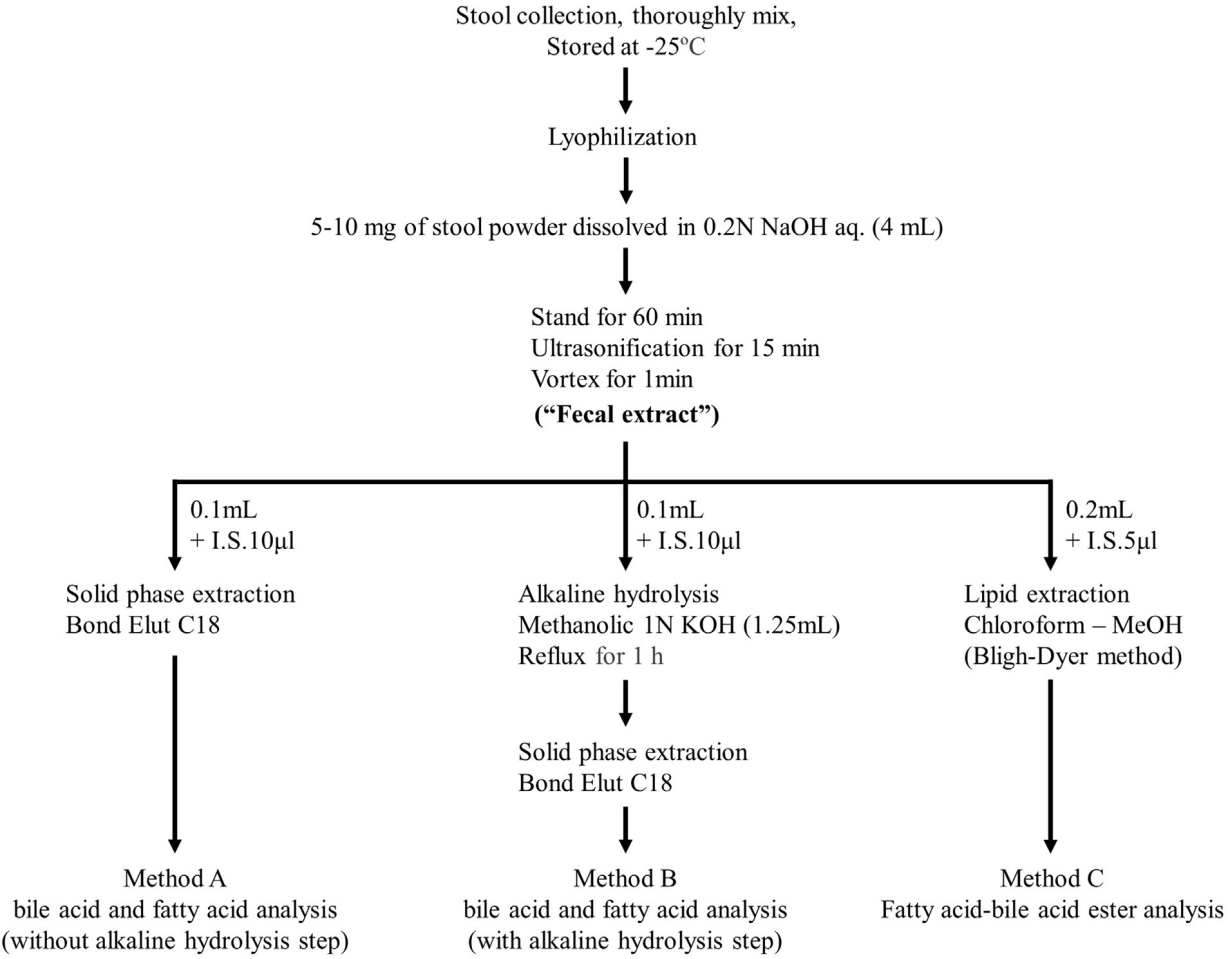
Sample preparation procedures for fecal BA and FA analyses with/without the alkaline hydrolysis. BA, bile acid; FA, fatty acid.

The key procedures are illustrated in Fig. 2. Lyophilized stool was thoroughly grounded to powder before use. To the powdered feces (5–10 mg, accurately weighed), 4 ml of 0.2 N NaOH solution was added. The suspension was allowed to stand at 40°C for 1 h and then sonicated for 15 min (“fecal extract”). The fecal extract was processed in the following three ways.

#### Method A

An aliquot (0.1 ml) of the fecal extract was transferred to a glass tube and diluted with 2 ml of distilled water. After adding 10 μl of IS solution, the mixture was applied to a Bond Elut C18 solid-phase extraction cartridge (500 mg/6 ml) that was preconditioned with 5 ml of methanol and 15 ml of H_2_O. The column was successively washed with 5 ml of H_2_O, and then BAs and FAs were eluted with 4 ml of methanol. After evaporation of the solvent under reduced pressure, the residue was dissolved in 1 ml of 60% methanol. A 5 μl or 1 μl aliquot was injected into the LC/MS instrument for BA or FA analysis, respectively.

#### Method B

Fecal extract (0.1 ml) was transferred to a reaction tube, and methanol (1.1 ml), 8 N KOH (150 μl), and 10 μl of IS (10 μl) were added. The mixture was refluxed for 1 h. After 9 ml of H_2_O was added, the reaction mixture was applied to a Bond Elut C18 column as described in Method A. Of note, 8% methanol was used for washing the column instead of water. The collected effluents were evaporated under reduced pressure. The residue was dissolved in 1 ml of 60% methanol. A 5 μl or 1 μl aliquot was injected into the LC/MS instrument for BA analysis or FA analysis, respectively.

#### Method C

Fecal extract (0.2 ml) was combined with 10 μl of IS solution and treated by the Bligh-Dyer’s extraction method: To a mixture of fecal extract and IS, 0.2 ml of water, 1.0 ml of methanol, and 0.5 ml of chloroform were added and thoroughly vortexed for 1 min. Subsequently, 0.5 ml of chloroform and 0.5 ml of water were added, and the mixture was vortexed for an additional 20 s. To clearly separate the phases, the mixture was centrifuged at 3,000 rpm for 10 min, and the lower layer was transferred to a glass tube. After the solvent was evaporated under reduced pressure, the residue was dissolved in 0.5 ml of 80% methanol, and a 5 μl aliquot was injected into the LC/MS instrument.

### LC/MS analysis

An LCMS-8050 tandem mass spectrometer equipped with an ESI probe and Nexera X2 ultra-high-pressure liquid chromatography system (Shimadzu Co, Kyoto, Japan) was used for the following runs:

#### BA analysis (condition I)

BA analysis was performed according to our previous method (24). An InertSustain C18 (150 mm × 2.1 mm internal diameter, 3 μm particle size; GL Sciences Inc., Tokyo, Japan) was employed at 40°C. A mixture of 5 mM ammonium acetate and acetonitrile was used as the eluent at a flow rate of 0.2 ml/min. Gradient elution was programmed as follows: ammonium acetate-acetonitrile (82:18, v/v) for 0.5 min, (78:22, v/v) for 0.5–2 min, (72:28, v/v) for 2–23 min, (62:38, v/v) for 23–33 min, (46:54, v/v) for 33–49 min, (2:98, v/v) for 49–58 min, (2:98, v/v) for 58–62 min, (82:18, v/v) for 62–62.1 min, and (82:18, v/v) for 62.1–68 min. Total run time was 68 min. The following MS condition was used: spray voltage, 3,000 V; heating block temperature, 400°C; nebulizing gas flow, 3 l/min; drying gas flow, 10 l/min; heating gas flow, 10 l/min; interface temperature, 300°C; collision gas (argon) pressure, 270 kPa; collision energy, 10–60 eV; all in the negative ion multiple reaction monitoring (MRM) mode. Data were analyzed using LabSolutions Insight LCMS software (Shimadzu). LC/MS parameters for each BA including retention time (RT), MRM transition, and collision energy were used according to a previous method (24).

#### BA analysis (condition II)

For saponified BA profiles, a SunShell C30 (100 mm × 2.1 mm ID, 2.6 μm particle size; ChromaNik Technologies Inc., Osaka, Japan) column was used at 40°C. A mixture of 5 mM ammonium acetate and acetonitrile was used as the eluent at a flow rate of 0.25 ml/min. Gradient elution was programmed as follows: ammonium acetate-acetonitrile (72:28, v/v) for 0.5 min, (66:34, v/v) for 0.5–5 min, (66:34, v/v) for 5–22 min, (62:38, v/v) for 22–38 min, (76:24, v/v) for 38–40 min, (95:5, v/v) for 40–44 min, (72:28, v/v) for 44–44.1 min, and (72:28, v/v) for 44.1–50 min. Total run time was 50 min. The same MS conditions as those for Condition I were used. LC/MS parameters for each BA are shown in supplemental Table S2. Note: Murideoxycholic acid, isoUDCA, 7-oxo-Δ^5^-3β-ol hyodeoxycholic acid, 3-oxo-CDCA, isoLCA, isoalloLCA, LCA, 3-oxo-alloLCA, 3-oxo-LCA, and *d*_4_-LCA hardly make fragmentation under normal collision-induced dissociation (CID). Therefore, we used selected ion monitoring mode for their quantitation.

#### FA analysis

An InertSustain C18 (150 mm × 2.1 mm ID, 3 μm particle size; GL Sciences Inc.) was employed at 40°C. A mixture of 5 mM ammonium acetate and methanol was used as the mobile phase, and the separation was carried out by linear gradient elution at a flow rate of 0.3 ml/min. The mobile phase composition was gradually changed as follows: ammonium acetate-methanol (12:88, v/v) for 1 min, (2:98, v/v) for 1–10 min, (2:98, v/v) for 10–14 min, (2:98, v/v) for 10–14 min, (12:88, v/v) for 14–14.1 min, and (12:88, v/v) for 14.1–20 min. The total run time was 20 min. The same MS conditions as those for Condition I were used. LC/MS parameters for each FA are shown in supplemental Table S3.

#### FA-isoBA analysis (intact analysis)

A separation column, InertSustain C8 (150 mm × 2.1 mm ID, 3 μm particle size; GL Sciences Inc.) was employed at 40°C. A mixture of pH 7, 5 mM ammonium acetate, and methanol was used as mobile phase, and the separation was carried out by linear gradient elution at a flow rate of 0.2 ml/min. Gradient elution was programmed as follows: ammonium acetate-methanol (16:84, v/v) for 0.5 min, (10:90, v/v) for 0.5–4 min, (2:98, v/v) for 4–12 min, (2:98, v/v) for 12–22 min, (10:90, v/v) for 22–22.1 min, and (10:90, v/v) for 22.1–28 min. Total run time was 28 min. The same MS parameters as those for Condition I were used. MRM data for each FA-isoBA are shown in Table 1.

**Table 1:** LC/MS parameters for the fatty acid bile acid esters exanimated in this study

Fatty acid bile acid ester	RT (min)	Precursor ion (*m/z*)	Product ion (*m/z*)	CE (eV)	Linear expression	*r*	LOD (nmol/g)	LOQ (nmol/g)
Ac-isoLCA	7.82	417.4	357.3	33	y = 1.51877x + 0.0002432	0.99938	0.39	1.31
Pam-isoLCA	17.44	613.7	357.3	43	y = 6.35702x + 0.0111798	0.99991	0.07	0.25
Ste-isoLCA	18.94	641.7	357.3	43	y = 5.32768x + 0.0291555	0.99987	0.08	0.28
Ole-isoLCA	17.75	639.7	357.3	45	y = 9.20428x + 0.0135359	0.99985	0.12	0.40
Lin-isoLCA	16.82	637.7	357.3	44	y = 8.31027x + 0.0065879	0.99993	0.16	0.54
Ac-isoDCA	5.69	433.4	373.3	31	y = 2.04851x + 0.00002836	0.99974	0.95	2.75
Pam-isoDCA	14.6	629.7	237.3	48	y = 6.25057x + 0.00241432	0.99999	0.16	0.52
Ste-isoDCA	15.84	657.7	265.4	47	y = 7.59746x + 0.0165732	0.99997	0.12	0.39
Ole-isoDCA	14.88	655.7	263.2	46	y = 3.13519x − 0.0009827	0.99993	0.12	0.38
Lin-isoDCA	14.06	653.7	261.4	41	y = 1.73655x + 0.0014496	0.99984	0.19	0.63
^13^C_18_-Ste-isoLCA	18.92	659.6	357.2	47				
^13^C_18_-Ste-isoDCA	15.82	675.6	345.2	52				

Abbreviations of each fatty acid-conjugated isobile acid are shown in Fig. 1.

LOD, limit of detection; LOQ, limit of quantification.

### Method validation

Method validation was performed according to the Validation of Analytical Procedures: text and methodology Q2(R1) guidelines (32). The linearity was determined by analyzing seven-point calibration standard solutions. For inter-day and intra-day validation of the method for the FA-isoBA analysis, 1 ml of the Bligh-Dyer extract was spiked with known amounts of FA-isoBAs mixtures at concentrations of 0.005, 0.05, and 0.5 μmol/g. For recovery tests, lyophilized stool was spiked with FA-isoBAs mixtures at concentrations of 0.005, 0.05, and 0.5 μmol/g. The spiked stool was processed with the same procedure as in Method C. The quantitation data were produced in quadruplicate. The lower LOD and the lower limit of quantification (LOQ) were determined by comparing signal-to-noise (S/N) ratios of 3 and 10, respectively. LOD and LOQ were obtained in the absence of matrix. Matrix effects were determined by comparing the linearity of the working solutions prepared from the extracted fecal samples according to the comparative study of Matuszewski *et al.* (33).

### Statistics

All data are reported as means ± SD in the tables and as means ± SEM in the figures. One-way ANOVA was used to determine the significance of differences between groups. Values of *P* < 0.05 were accepted as statistically significant.

### Ethical considerations

This study was approved by the Institutional Review Board of Juntendo University (approval number 19–184). Informed consent was obtained from each subject or the parents of each subject prior to enrollment in the study. This study followed the 1964 Helsinki Declaration and its later amendments or with comparable ethical standards (as revised in Edinburgh in 2000).

## Results

### Fecal bile acid and FA concentrations with/without alkaline hydrolysis

**Fig. 3:**
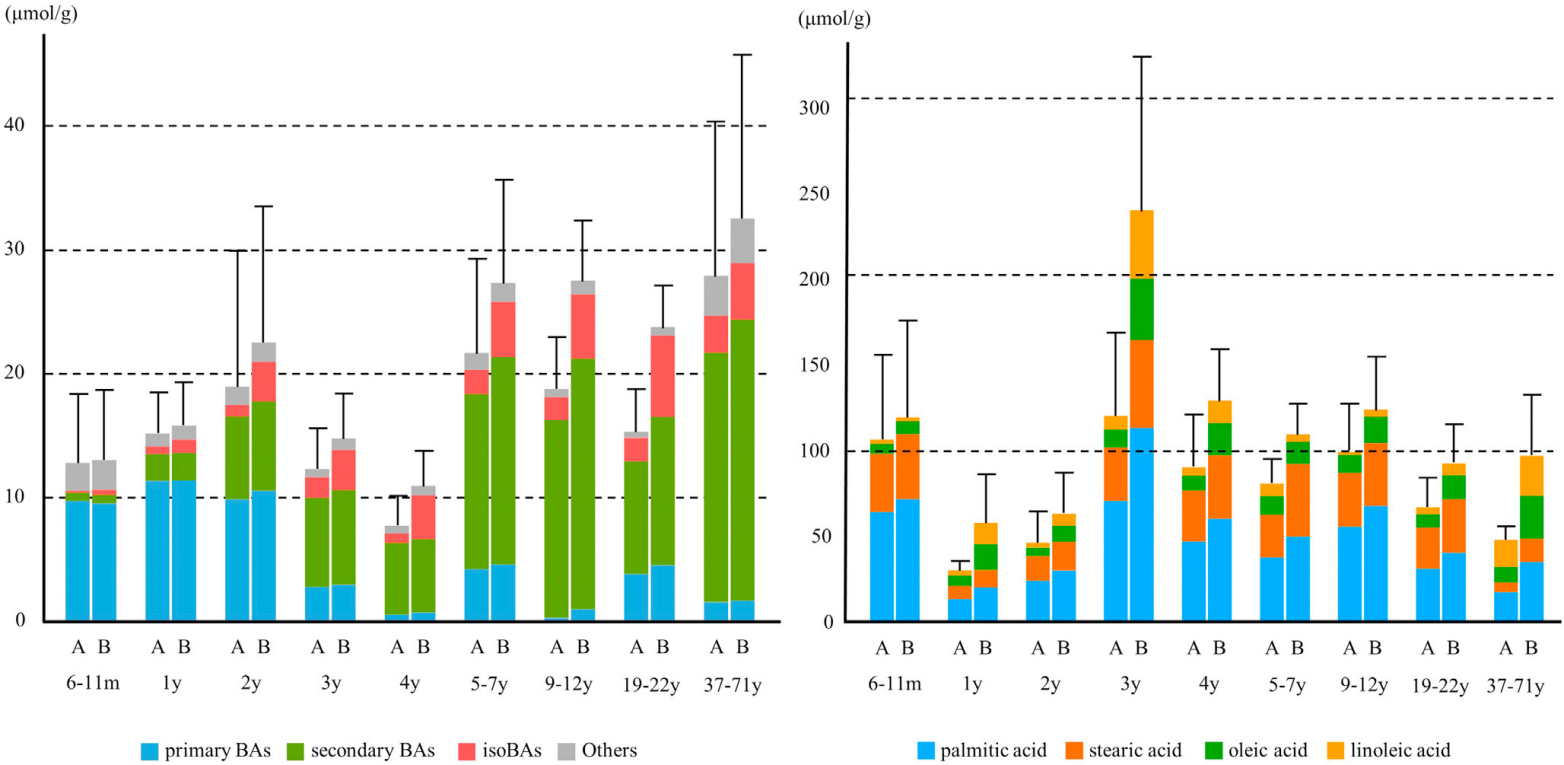
Fecal BA (left panel) and FA (right panel) concentrations obtained by Methods A and B. BA, bile acid; m, months; y, years; FA, fatty acid.

Figure 3 presents the fecal BA and FA concentrations obtained by Methods A and B. Long-chain FA-conjugated BA has highly hydrophobic properties and is expected to resist saponification by mild alkaline treatment. We refluxed the fecal extract in 1M methanol KOH for 1 h (Method B). Of note, we examined stability of each BA and found that some BA species can be partially decomposed under this condition. These BAs (decomposed ≥5%) are described in supplemental Table S4. With the described treatment, we found 2–208% higher total bile acids (TBAs) than those without the treatment (Method A). TBA plateaued under the described conditions. Higher temperatures or prolonged incubation time did not further elevate these concentrations. FA analysis showed that long-chain FAs including Pam, Ste, Ole, and Lin acids were predominantly found, and their concentrations also plateaued with the described alkaline treatment. The data shown in Table 2 compare individual BAs obtained by Methods A and B. Other forms of BAs including amino acylated (e.g., glycine or taurine), sulfated, and double-conjugated (e.g., glycine-sulfated) BAs did not change with/without the alkaline treatment. Thus, these conjugated BAs did not account for the elevation of TBA with the alkaline treatment. Interestingly, BAs having 3β-hydroxy group (isoBAs) such as isoCDCA, isoUDCA, isoDCA, isoLCA, 12β-OH-isoLCA, and 12-oxo-isoLCA were markedly elevated with the alkaline treatment. In adult feces (19–22 years, n = 14), up to 42.1% (2.2–207.8%) higher concentrations were found than those without the treatment. These data indicate that a considerable portion of fecal isoBAs are present in saponifiable forms, presumably long-chain FAs through 3β-acyl bonds. Of note, much less trihydroxy isoBAs (e.g., isoCA and isoUCA) were saponifiable than monohydroxy and dihydroxy isoBAs. These observations suggest that FA conjugation preferably occurs in monohydroxy and dihydroxy isoBAs.

**Table 2:** Fecal BA concentrations in by age groups obtained by Methods A and B

(μmol/g)	6–11 months (N=6)	1 year (N=6)	2 years (N=7)	3 years (N=7)	4 years (N=7)	5–7 years (N=7)	9–12 years (N=6)	19–22 years (N=14)	37–71 years (N=4)
	Mean ± SD	Mean ± SD	Mean ± SD	Mean ± SD	Mean ± SD	Mean ± SD	Mean ± SD	Mean ± SD	Mean ± SD
TBA (Method A)	14.72 ± 13.31	16.90 ± 8.01	20.14 ± 27.21	14.32 ± 8.07	9.50 ± 5.87	24.23 ± 17.19	21.80 ± 9.48	20.04 ± 13.17	30.72 ± 21.87
TBA (Method B)	14.77 ± 12.62	17.46 ± 8.00	23.81 ± 26.99	16.56 ± 9.26	12.83 ± 7.11	30.03 ± 18.88	30.62 ± 11.17	28.02 ± 12.83	35.11 ± 23.15
CA (Method A)	6.86 ± 7.88	8.58 ± 5.54	5.26 ± 11.45	2.17 ± 1.91	0.40 ± 0.64	3.40 ± 7.10	0.17 ± 0.23	2.72 ± 3.91	1.06 ± 1.66
CA (Method B)	6.66 ± 7.60	8.52 ± 5.69	5.63 ± 12.04	2.25 ± 1.80	0.51 ± 0.75	3.58 ± 7.02	0.59 ± 0.45	3.13 ± 4.08	1.08 ± 1.58
CDCA (Method A)	2.87 ± 3.60	2.79 ± 1.98	4.62 ± 9.95	0.64 ± 0.57	0.16 ± 0.16	0.84 ± 1.43	0.14 ± 0.15	1.13 ± 1.26	0.52 ± 0.54
CDCA (Method B)	2.88 ± 3.52	2.86 ± 2.06	4.95 ± 10.55	0.75 ± 0.58	0.22 ± 0.17	1.04 ± 1.54	0.43 ± 0.32	1.43 ± 1.31	0.64 ± 0.66
UDCA (Method A)	0.24 ± 0.50	0.63 ± 0.40	2.29 ± 3.61	0.88 ± 0.70	0.31 ± 0.35	1.42 ± 2.71	0.23 ± 0.20	1.18 ± 1.39	1.08 ± 1.06
UDCA (Method B)	0.24 ± 0.51	0.66 ± 0.42	2.47 ± 3.87	0.97 ± 0.78	0.38 ± 0.35	1.52 ± 2.77	0.48 ± 0.38	1.43 ± 1.53	1.16 ± 1.16
DCA (Method A)	0.00 ± 0.00	0.04 ± 0.08	2.19 ± 2.76	2.85 ± 3.99	3.00 ± 2.90	6.06 ± 5.96	8.92 ± 5.22	3.10 ± 2.53	12.77 ± 9.58
DCA (Method B)	0.00 ± 0.00	0.04 ± 0.09	2.39 ± 2.91	3.00 ± 4.11	3.01 ± 2.75	7.95 ± 7.47	12.28 ± 5.59	4.73 ± 3.79	14.47 ± 10.29
LCA (Method A)	0.00 ± 0.00	0.00 ± 0.01	0.94 ± 0.70	1.27 ± 1.45	1.40 ± 1.01	2.85 ± 2.79	5.26 ± 2.75	1.85 ± 1.67	3.80 ± 2.51
LCA (Method B)	0.00 ± 0.00	0.01 ± 0.01	1.00 ± 0.74	1.40 ± 1.59	1.44 ± 0.99	3.19 ± 3.29	5.62 ± 3.01	2.08 ± 1.83	4.42 ± 3.27
UCA (Method A)	0.46 ± 0.93	1.50 ± 1.59	1.00 ± 1.43	1.91 ± 3.31	0.67 ± 1.16	1.73 ± 3.32	0.14 ± 0.09	2.30 ± 5.00	0.50 ± 0.45
UCA (Method B)	0.45 ± 0.93	1.53 ± 1.59	1.11 ± 1.51	1.94 ± 3.30	0.70 ± 1.21	1.95 ± 3.30	0.35 ± 0.20	2.97 ± 5.81	0.53 ± 0.48
12β-OH-LCA (Method A)	0.00 ± 0.00	0.01 ± 0.02	0.26 ± 0.24	0.31 ± 0.47	0.43 ± 0.44	2.10 ± 2.85	1.45 ± 1.29	0.68 ± 0.78	2.01 ± 2.22
12β-OH-LCA (Method B)	0.00 ± 0.00	0.01 ± 0.02	0.26 ± 0.24	0.33 ± 0.51	0.41 ± 0.42	2.15 ± 2.88	1.53 ± 1.30	0.77 ± 0.77	2.08 ± 2.35
12-oxo-LCA (Method A)	0.00 ± 0.00	0.00 ± 0.01	0.18 ± 0.12	0.43 ± 0.61	0.83 ± 0.99	1.07 ± 1.32	1.84 ± 0.95	2.23 ± 2.85	1.43 ± 0.80
12-oxo-LCA (Method B)	0.00 ± 0.00	0.00 ± 0.01	0.19 ± 0.12	0.46 ± 0.67	0.80 ± 0.90	1.11 ± 1.36	1.89 ± 1.01	2.26 ± 2.74	1.48 ± 0.88
isoCA (Method A)	0.05 ± 0.05	0.28 ± 0.42	0.08 ± 0.12	0.21 ± 0.19	0.03 ± 0.04	0.20 ± 0.38	0.01 ± 0.02	0.29 ± 0.45	0.14 ± 0.22
isoCA (Method B)	0.06 ± 0.05	0.30 ± 0.41	0.11 ± 0.11	0.26 ± 0.22	0.07 ± 0.06	0.25 ± 0.43	0.08 ± 0.04	0.36 ± 0.47	0.15 ± 0.20
isoCDCA (Method A)	0.02 ± 0.03	0.17 ± 0.25	0.13 ± 0.17	0.09 ± 0.08	0.03 ± 0.03	0.09 ± 0.14	0.01 ± 0.01	0.15 ± 0.18	0.05 ± 0.04
isoCDCA (Method B)	0.26 ± 0.46	0.45 ± 0.33	0.64 ± 0.33	0.76 ± 0.91	0.44 ± 0.21	0.56 ± 0.37	0.71 ± 0.42	0.70 ± 0.27	0.10 ± 0.04
isoUDCA (Method A)	0.04 ± 0.09	0.05 ± 0.09	0.17 ± 0.33	0.23 ± 0.19	0.06 ± 0.06	0.23 ± 0.36	0.06 ± 0.05	0.25 ± 0.32	0.26 ± 0.29
isoUDCA (Method B)	0.05 ± 0.12	0.20 ± 0.34	0.43 ± 0.31	0.76 ± 0.86	0.30 ± 0.16	0.34 ± 0.42	0.44 ± 0.31	0.63 ± 0.38	0.31 ± 0.31
isoDCA (Method A)	0.00 ± 0.00	0.00 ± 0.00	0.20 ± 0.24	0.37 ± 0.59	0.24 ± 0.26	0.51 ± 0.46	0.69 ± 0.39	0.31 ± 0.22	1.44 ± 1.08
isoDCA (Method B)	0.00 ± 0.00	0.03 ± 0.06	1.10 ± 1.01	0.60 ± 0.79	1.53 ± 1.33	1.33 ± 0.92	1.97 ± 0.54	2.22 ± 1.65	2.21 ± 1.01
isoLCA (Method A)	0.00 ± 0.00	0.00 ± 0.00	0.21 ± 0.18	0.29 ± 0.34	0.27 ± 0.20	0.58 ± 0.58	0.93 ± 0.41	0.41 ± 0.39	0.75 ± 0.65
isoLCA (Method B)	0.00 ± 0.00	0.00 ± 0.01	0.64 ± 0.63	0.39 ± 0.45	1.03 ± 0.85	1.50 ± 1.75	1.75 ± 0.59	1.94 ± 1.85	1.31 ± 0.88
isoUCA (Method A)	0.05 ± 0.12	0.11 ± 0.23	0.14 ± 0.20	0.44 ± 0.64	0.10 ± 0.15	0.27 ± 0.36	0.05 ± 0.05	0.44 ± 0.96	0.18 ± 0.16
isoUCA (Method B)	0.05 ± 0.12	0.11 ± 0.24	0.15 ± 0.21	0.44 ± 0.63	0.10 ± 0.15	0.28 ± 0.38	0.06 ± 0.06	0.47 ± 1.03	0.18 ± 0.16
12β-OH-isoLCA (Method A)	0.00 ± 0.00	0.00 ± 0.00	0.01 ± 0.03	0.02 ± 0.03	0.02 ± 0.02	0.10 ± 0.10	0.09 ± 0.08	0.05 ± 0.05	0.16 ± 0.17
12β-OH-isoLCA (Method B)	0.00 ± 0.00	0.00 ± 0.00	0.12 ± 0.15	0.02 ± 0.04	0.09 ± 0.10	0.20 ± 0.19	0.17 ± 0.12	0.25 ± 0.26	0.32 ± 0.22
12-oxo-isoLCA (Method A)	0.00 ± 0.00	0.00 ± 0.00	0.07 ± 0.05	0.12 ± 0.16	0.17 ± 0.18	0.20 ± 0.20	0.30 ± 0.15	0.32 ± 0.33	0.34 ± 0.19
12-oxo-isoLCA (Method B)	0.00 ± 0.00	0.00 ± 0.01	0.28 ± 0.25	0.16 ± 0.19	0.56 ± 0.67	0.42 ± 0.39	0.65 ± 0.37	0.93 ± 0.75	0.49 ± 0.08
oxo-BAs^a^ (Method A)	1.89 ± 1.74	1.68 ± 1.52	0.90 ± 1.14	1.44 ± 0.35	0.74 ± 0.31	1.24 ± 1.29	0.87 ± 0.57	2.14 ± 1.12	1.01 ± 0.77
oxo-BAs^a^ (Method B)	1.72 ± 1.70	1.61 ± 1.45	0.77 ± 1.07	1.13 ± 0.24	0.49 ± 0.23	1.11 ± 1.27	0.54 ± 0.41	1.03 ± 0.93	0.60 ± 0.63
3-oxo-Δ4-BAs^b^ (Method A)	0.00 ± 0.00	0.00 ± 0.00	0.00 ± 0.00	0.01 ± 0.02	0.00 ± 0.01	0.01 ± 0.02	0.00 ± 0.00	0.02 ± 0.03	0.00 ± 0.00
3-oxo-Δ4-BAs^b^ (Method B)	0.00 ± 0.00	0.00 ± 0.00	0.00 ± 0.00	0.00 ± 0.00	0.00 ± 0.00	0.01 ± 0.02	0.00 ± 0.00	0.00 ± 0.00	0.00 ± 0.00
BA-3S^c^ (Method A)	1.52 ± 1.12	0.60 ± 0.68	0.58 ± 1.34	0.19 ± 0.47	0.48 ± 1.18	0.75 ± 1.13	0.46 ± 1.03	0.11 ± 0.36	2.59 ± 1.66
BA-3S^c^ (Method B)	1.60 ± 1.17	0.63 ± 0.67	0.70 ± 1.57	0.23 ± 0.56	0.44 ± 1.09	0.75 ± 1.13	0.45 ± 1.01	0.15 ± 0.43	2.83 ± 1.78
others (Method A)	0.70 ± 0.32	0.46 ± 0.22	0.89 ± 1.81	0.47 ± 0.59	0.15 ± 0.11	0.61 ± 0.90	0.19 ± 0.16	0.39 ± 0.50	0.63 ± 0.63
others (Method B)	0.80 ± 0.28	0.50 ± 0.20	0.88 ± 1.46	0.71 ± 0.66	0.30 ± 0.15	0.80 ± 0.83	0.66 ± 0.38	0.53 ± 0.39	0.76 ± 0.55

Abbreviations of each BA are shown in Supplemental Table 1.

SD, standard deviation; 3S, 3-sulfate; Unit: μmol/g; Values are expressed as mean ± SD.

a oxo-BAs is the sum of 3-oxo-CA, 3-oxo-CDCA, 3-oxo-UDCA, 3-oxo-DCA, 3-oxo-LCA, 7-oxo-DCA, 12-oxo-CDCA, 7-oxo-LCA, 3-oxo-alloCDCA, 3-oxo-alloDCA, and 3-oxo-alloLCA.

b 3-oxo-Δ4-BAs is the sum of CA-Δ4-3-one, GCA-Δ4-3-one, TCA-Δ4-3-one, CDCA-Δ4-3-one, GCDCA-Δ4-3-one, TCDCA-Δ4-3-one, CA-Δ4,6-3-one, and CDCA-Δ4,6-3-one.

c BA-3S is the sum of CA-3S, GCA-3S, TCA-3S, C CDCA-3S, GCDCA-3S, TCDCA-3S, UDCA-3S, GUDCA-3S, TUDCA-3S, DCA-3S, GDCA-3S, TDCA-3S, LCA-3S, GLCA-3S, and TLCA-3S.

### Effect of concentrated alkaline treatment on the epimerization of hydroxy group

It is possible to cause α-β epimerization of hydroxyl groups at the C3-position during the strong alkaline treatment. To test for possible epimerization, synthetic FA-isoBAs were subjected to the described alkaline hydrolysis (Method B), and the generated free BAs were determined by LC-MS (supplemental Table S5). All FA-conjugated BAs having 3β-hydroxy groups (FA-isoBAs) quantitatively produced their corresponding 3β-hydroxy BAs (isoLCA and isoDCA). However, FA-conjugated 3α-hydroxy BAs, Ac-LCA and Ste-LCA, produced their 3β-epimer (isoLCA) at 20% and 32%, respectively. Interestingly, Ste-DCA produced 100% DCA, and no epimerized product (isoDCA) was generated.

### Measurement of saponifiable bile acids in feces from different age groups

Saponifiable BAs were almost undetectable in specimens from 6–11-month-old subjects (Table 2). Two primary BAs, CA and CDCA, accounted for 70.6% (33.4–94.4%) of fecal TBA. Meanwhile, secondary BAs (i.e., isoCDCA and isoCA) accounted for 24.1% (3.5–64.7%), but DCA and LCA were not detected in this age group. Saponifiable BAs were first detected in specimens from 1-year-olds at a low concentration (0.56 μmol/g), which accounted for 3.2% (0.0–16.3%) of TBA. This concentration was markedly elevated in the 2-years age group (3.7 μmol/g; 1.5–7.0 μmol/g, 15.4%; 3.7–40.8% of TBA) when large secondary BAs simultaneously appeared. IsoDCA and isoLCA were also first found in this age group. Once the gut microbiome was established after 2 years, saponifiable BAs were consistently excreted throughout life, which accounted for 10–30% of total fecal BA. The predominant saponifiable BAs found in all age groups were isoDCA and isoLCA and isoDCA consistently present at slightly higher amounts than isoLCA.

### Estimation of FA-conjugated bile acids by precursor ion scanning

**Fig. 4:**
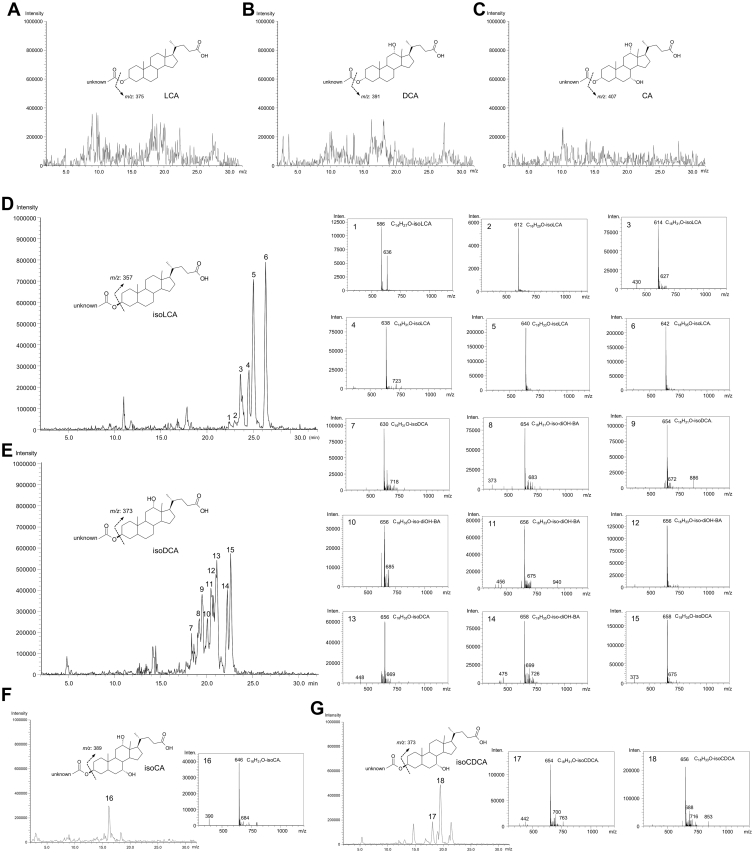
Precursor ion scanned data of the representative fecal specimens: MS chromatograms were obtained from targeting BA products generated by collision-induced dissociation. Precursor ion spectra were extracted from individual peaks obtained from the LC effluent. Representative spectra from adult fecal specimen were shown except panel G (Panel G: A 6-months infant specimen). A: Precursor ion scan chromatograms targeting LCA product ion of m/z 375, B: Precursor ion scan chromatograms targeting DCA (12β-OH-LCA, CDCA, UDCA) product ion of m/z 391, C: Precursor ion scan chromatograms targeting CA (UCA) product ion of m/z 407, D: Precursor ion scan chromatograms targeting isoLCA product ion of m/z 357, E: Precursor ion scan chromatograms targeting isoDCA (12β-OH-isoLCA, isoCDCA, isoUDCA) product ion of m/z 373, F: Precursor ion scan chromatograms targeting isoCA (isoUCA) product ion of m/z 389, 1: The compound expected from the precursor ion spectra of m/z 586 is C_14_H_27_O-isoLCA, 2: The compound expected from the precursor ion spectra of m/z 612 is C_16_H_29_O-isoLCA, 3: The compound expected from the precursor ion spectra of m/z 614 is C_16_H_31_O-isoLCA, 4: The compound expected from the precursor ion spectra of m/z 638 is C_18_H_31_O-isoLCA, 5: The compound expected from the precursor ion spectra of m/z 640 is C_18_H_33_O-isoLCA, 6: The compound expected from the precursor ion spectra of m/z 642 is C_18_H_35_O-isoLCA, 7: The compound expected from the precursor ion spectra of m/z 630 is C_16_H_31_O-isoDCA, 8: The compound expected from the precursor ion spectra of m/z 654 is C_18_H_31_O-12β-OH-isoLCA (isoCDCA, isoUDCA), 9: The compound expected from the precursor ion spectra of m/z 654 is C_18_H_31_O-isoDCA, 10–12: The compound expected from the precursor ion spectra of m/z 656 is 12β-OH-isoLCA (isoCDCA, isoUDCA), 13: The compound expected from the precursor ion spectra of 658: m/z 656 is C_18_H_33_O- C_18_H_33_O-isoDCA, 14: The compound expected from the precursor ion spectra of m/z 658 is C_18_H_35_O-12β-OH-isoLCA, 15: The compound expected from the precursor ion spectra of m/z 658 is C_18_H_35_O-isoDCA, 16: The compound expected from the precursor ion spectra of m/z 646 is C_16_H_31_O-isoCA, 17: The compound expected from the precursor ion spectra of m/z 654 is C_18_H_31_O-isoCDCA, 18: The compound expected from the precursor ion spectra of m/z 656 is C_18_H_33_O-isoCDCA. BA, bile acid; LCA, lithocholic acid; DCA, deoxycholic acid; 12β-OH-LCA, 3α,12β-dihydroxy-5β-cholanoic acid; CDCA, chenodeoxycholic acid; UDCA, ursodeoxycholic acid; CA, cholic acid; UCA, ursocholic acid; 12β-OH-isoLCA, 3β,12β-dihydroxy-5β-cholanoic acid.

To estimate the structure of FA-BAs in feces, we performed the precursor ion scanning in LC/MS. Based on the fragmentation patterns of authentic Ole-DCA, Ste-DCA, Ole-isoDCA, Ste-isoDCA, Ole-LCA, Ste-LCA Ole-isoLCA, and Ste-isoLCA, we determined product (daughter) ions, [BA−H_2_O−H]^−^ and [BA−H]^−^ for FA-3α-hydroxy BAs; and [BA−H_2_O−H]^−^ for FA-isoBA, respectively (supplemental Figure S1). Precursor ions (*m/z*) from the respective daughter ions were monitored. Figure 4 presents representative precursor ion scan spectra of the Bligh-Dyer extract from adult and infant specimens. No precursor ions corresponding to the FA conjugates were found for product ions m/z 375 (LCA) (Panel A), 391 (DCA or other dihydroxy bile acids) (Panel B), 407 (CA or UCA) (Panel C). Therefore, FA-esterified CA, UCA, DCA, and LCA were not expected to present. When the product ion of *m/z* 357 (isoLCA) was selected (Panel D), possible precursor ions of *m/z* 614 (C_16_H_31_O-isoLCA), 638 (C_18_H_31_O-isoLCA), 640 (C_18_H_33_O-isoLCA), and 642 (C_18_H_35_O-isoLCA) were identified. Similarly, when the product ion of *m/z* 373 (isoDCA, isoCDCA, or isoUDCA) was selected (Panel E), precursor ions of *m/z* 630 (C_16_H_31_O-isoDCA), 654 (C_18_H_31_O-isoDCA), 656 (C_18_H_33_O-isoDCA), and 658 (C_18_H_35_O-isoDCA) were identified. Molecular formulas C_16_H_31_O, C_18_H_31_O, C_18_H_33_O, and C_18_H_35_O correspond to Pam, Ste, Lin, and Ole, respectively. The precursor scan spectra obtained from *m/z* 389 (isoCA) (Panel F) as a product ion suggested the presence of small amounts of esterified isoCA (Peak 16). These data were consistent with those obtained with alkaline hydrolysis (Methods A, B). Of note, we additionally analyzed fecal extracts from three infants (6-month-old, 7-month-old, and 1-year-old, all male) whose stool detected isoCDCA but did not detect DCA and LCA by the alkaline hydrolysis method. The parental ion scan data based on *m/z* 373 as a product ion (Panel G) indicated the possible presence of FA-isoCDCA (or isoUDCA) esters (Peaks 17 and 18).

### LC-MS settings for intact FA-isoBA analysis

**Fig. 5:**
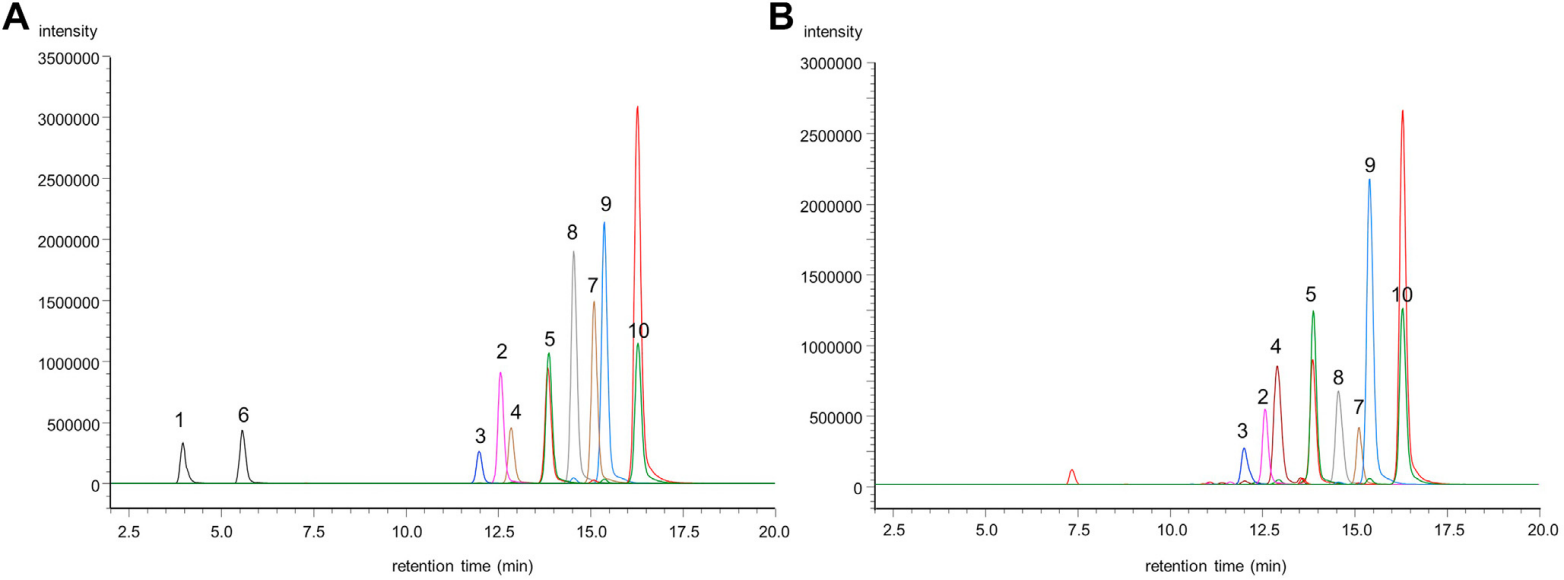
LC/MS chromatograms of fatty acid bile acid esters. A: Typical chromatogram of ten variants of standards (concentration: 300 pmol/ml) and internal standards, B: A representative chromatogram of human adult fecal extract, 1: Ac-isoDCA (433.4 > 373.3), 2: Pam-isoDCA (629.7 > 237.3), 3: Lin-isoDCA (653.7 > 261.4), 4: Ole-isoDCA (655.7 > 263.3), 5: Ste-isoDCA (657.7 > 265.3), 6: Ac-isoLCA (417.4 > 357.3), 7: Pam-isoLCA (613.7 > 357.3), 8: Lin-isoLCA (637.7 > 357.3), 9: Ole-isoLCA (639.7 > 357.3), 10: Ste-isoLCA (641.7 > 357.3). The red chromatograms of No. 5 and No. 10 represent internal standards. isoDCA, isodeoxycholic acid; isoLCA, isolithocholic acid; Pam, palmitic acid; Ste, stearic acid; Ole, oleic acid; Lin, linoleic acid; Ac-isoLCA, 3β-acetyl isolithocholic acid.

Intact FA-isoBAs were analyzed by ESI-MS/MS operating in the negative-ion mode. FA-isoBAs were readily ionized by the ESI and fragmented under the condition of low-energy CID, in which a deprotonated ion [M-H]^−^ was observed as the base peak. The CID spectra of this ion gave a dehydrated molecule [isoBA−H_2_O]^–^ as the major fragment from all FA-isoBAs. Therefore, *m/z* 357.3 [isoLCA−H_2_O]^−^ was chosen for the product ion of FA-isoLCA. However, *m/z* 373.3 [isoDCA−H_2_O]^−^ overlapped the fecal matrix component at close retention times for most FA-isoDCAs (except for Ac-isoDCA). Therefore, [FA−H_2_O]^−^ was selected for the product ion of FA-isoDCA. Ten FA-isoBAs and ISs were well separated on an InertSustain C8 (150 mm × 2.1 mm ID, 3 μm particle size) column with ammonium acetate-buffered aqueous methanol as the mobile phase. Retention time, MRM transition, and collision energy for each FA-isoBA examined in this study are presented in Table 1. Figure 5 (left panel) presents a typical MRM chromatogram of 10 authentic standards of FA-isoBAs. A typical chromatogram obtained from an adult fecal specimen is also presented in Fig. 5 (right panel).

## Method validation

The apparent linearity of the method was examined using the 10 FA-isoBA standard calibrators ranging from 1 to 1,000 pmol/ml (Table 1). Peak area ratios (analyte/IS) were proportional to the concentrations of all FA-isoBAs. The calibration curves appeared linear, and the slopes, intercepts, and correlation coefficients were determined by least square lines with a weighting factor of 1/(concentration)^2^. A satisfactory linearity, that is, a correlation coefficient (r) greater than 0.999, was obtained within the experimental concentration range for all 10 FA-isoBAs. The LOD as determined by a peak with an S/N ≥ 3 was 0.07–0.95 nmol/g. Similarly, LOQ as determined by an S/N ≥ 10 was 0.25–2.75 nmol/g.

Intra- and inter-day validation was performed using three different concentrations of the spiked samples (Table 3). Of note, the calibration curve was plotted in the absence of matrix; these are apparent recovery rates, which may not necessarily reflect endogenous material. The assay precision was determined by quadruplicate measurements per day, and inter-day precision was assessed over a 4-day period. The intra-day SD at three different concentrations was between 3.8–13.6%. The inter-day SD was 3.8–13.9% for all analytes at three concentrations.

**Table 3:** Intra-day and inter-day assay variability of FA-BA esters

FA-isoBA	Intra-day			Inter-day		
	5 nmol/g	50 nmol/g	500 nmol/g	5 nmol/g	50 nmol/g	500 nmol/g
	Mean ± SD	Mean ± SD	Mean ± SD	Mean ± SD	Mean ± SD	Mean ± SD
Ac-isoLCA	5.50 ± 0.24	64.1 ± 0.89	714 ± 50.2	5.80 ± 0.37	67.9 ± 8.15	695 ± 46.8
Pam-isoLCA	4.02 ± 0.01	55.5 ± 1.91	519 ± 18.5	4.36 ± 0.58	52.8 ± 4.68	503 ± 44.3
Ste-isoLCA	4.66 ± 0.18	50.6 ± 0.62	442 ± 8.1	4.70 ± 0.46	54.2 ± 3.27	478 ± 29.3
Ole-isoLCA	4.99 ± 0.02	55.9 ± 0.71	560 ± 13.3	5.11 ± 0.27	53.9 ± 2.05	565 ± 45.3
Lin-isoLCA	3.86 ± 0.04	55.8 ± 1.34	590 ± 2.5	4.32 ± 0.43	54.8 ± 3.67	535 ± 52.1
Ac-isoDCA	5.68 ± 0.36	55.7 ± 1.55	590 ± 1.5	5.27 ± 0.36	58.9 ± 3.38	613 ± 25.3
Pam-isoDCA	6.27 ± 0.28	65.0 ± 1.40	598 ± 6.3	5.86 ± 0.38	63.9 ± 4.61	593 ± 41.0
Ste-isoDCA	5.35 ± 0.08	57.1 ± 0.74	511 ± 8.0	5.16 ± 0.22	50.2 ± 2.02	450 ± 18.8
Ole-isoDCA	6.76 ± 0.06	58.3 ± 1.08	557 ± 8.2	5.89 ± 0.82	57.0 ± 7.48	565 ± 77.1
Lin-isoDCA	5.60 ± 0.43	60.3 ± 0.62	612 ± 7.0	5.24 ± 0.55	56.3 ± 5.43	567 ± 52.8

Abbreviations of each FA-conjugated isoBA are shown in Fig. 1.

FA, fatty acid; SD, standard deviation.

A recovery test was performed using adult lyophilized stool samples spiked with three different concentrations of FA-isoBAs (Table 4). Good recovery rates, 70.7–102.8%, were obtained for all long-chain FA-isoBAs at three concentrations. Meanwhile, the recovery rates of Ac-isoBAs were significantly lower at 5.6–60.5%. Particularly, poor recovery of 5.6–11.0% for Ac-isoDCA was unsatisfactory.

**Table 4:** Relative recoveries (%) of FA-BA esters from fecal specimens

FA-isoBA	0.005 μmol/g	0.05 μmol/g	0.5 μmol/g
	% ± SD	% ± SD	% ± SD
Ac-isoLCA	45.4 ± 11.8	60.5 ± 13.2	56.8 ± 13.8
Pam-isoLCA	76.0 ± 4.4	95.0 ± 2.5	102.8 ± 4.8
Ste-isoLCA	92.6 ± 10.0	98.5 ± 4.8	96.5 ± 1.5
Ole-isoLCA	75.9 ± 6.0	99.4 ± 2.3	85.6 ± 2.0
Lin-isoLCA	70.7 ± 6.5	96.7 ± 3.1	84.3 ± 3.1
Ac-isoDCA	11.0 ± 8.9	8.3 ± 2.3	5.6 ± 3.2
Pam-isoDCA	82.9 ± 3.0	89.2 ± 14.5	86.8 ± 10.3
Ste-isoDCA	73.2 ± 11.4	93.7 ± 8.7	85.8 ± 4.9
Ole-isoDCA	81.2 ± 7.0	87.7 ± 14.1	86.5 ± 8.5
Lin-isoDCA	83.0 ± 12.4	84.7 ± 10.2	79.0 ± 6.9

Abbreviations of each fatty acid-conjugated isobile acid are shown in Fig. 1.

FA, fatty acid; SD, standard deviation.

### Quantification of FA-isoBAs in different age groups

Fecal FA-isoBAs concentrations of 64 healthy volunteers from infants to 71 years of age are shown in Table 5. In agreement with the saponifiable BAs quantified by Methods A and B (Table 2), FA-isoBAs were slightly detectable in the feces of 6–11-month-olds. The small amounts of FA-isoBAs found in the 1-year-old group specimens (total, 0.03 μmol/g). This level was significantly elevated in the 2-year-old specimens (0.90 μmol/g). Although the total concentration in the 3-year-old group was lower (0.19 μmol/g) for unclear reason, it immediately returned to 1.15 μmol/g in the 4-year-olds. FA-isoBAs were consistently found (0.75–1.5 μmol/g) thereafter. In most age groups, FA-isoDCA was found at a slightly higher concentration than FA-isoLCA, although unconjugated isoDCA and isoLCA were present in essentially equal concentrations (See Table 2, Method A). These FA-isoBA concentrations were similar to those of saponifiable BAs estimated by Methods A and B (Table 2).

**Table 5:** Changes in fecal FA-conjugated isoBA concentrations by age groups

(μmol/g)	6–11 months (N=6)	1 year (N=6)	2 years (N=7)	3 years (N=7)	4 years (N=7)	5–7 years (N=7)	9–12 years (N=6)	19–22 years (N=14)	37–71 years (N=4)
	Mean ± SD	Mean ± SD	Mean ± SD	Mean ± SD	Mean ± SD	Mean ± SD	Mean ± SD	Mean ± SD	Mean ± SD
Total	0.001 ± 0.003	0.031 ± 0.063	0.895 ± 1.020	0.190 ± 0.309	1.148 ± 0.995	0.894 ± 0.812	1.071 ± 0.608	1.504 ± 1.451	0.756 ± 0.322
Ac-isoDCA	0.000 ± 0.000	0.000 ± 0.000	0.000 ± 0.000	0.000 ± 0.000	0.000 ± 0.000	0.000 ± 0.000	0.000 ± 0.000	0.000 ± 0.000	0.000 ± 0.000
Pam-isoDCA	0.000 ± 0.000	0.002 ± 0.004	0.184 ± 0.226	0.026 ± 0.051	0.212 ± 0.277	0.087 ± 0.074	0.178 ± 0.144	0.170 ± 0.195	0.094 ± 0.061
Ste-isoDCA	0.000 ± 0.000	0.014 ± 0.030	0.229 ± 0.358	0.023 ± 0.036	0.205 ± 0.212	0.096 ± 0.084	0.179 ± 0.161	0.166 ± 0.151	0.086 ± 0.018
Ole-isoDCA	0.000 ± 0.001	0.011 ± 0.023	0.177 ± 0.181	0.063 ± 0.108	0.259 ± 0.185	0.192 ± 0.147	0.259 ± 0.121	0.331 ± 0.250	0.177 ± 0.051
Lin-isoDCA	0.000 ± 0.001	0.002 ± 0.004	0.099 ± 0.106	0.043 ± 0.068	0.125 ± 0.093	0.113 ± 0.094	0.135 ± 0.084	0.182 ± 0.155	0.048 ± 0.019
Ac-isoLCA	0.000 ± 0.000	0.000 ± 0.000	0.004 ± 0.005	0.000 ± 0.001	0.014 ± 0.017	0.002 ± 0.002	0.005 ± 0.004	0.020 ± 0.045	0.002 ± 0.002
Pam-isoLCA	0.000 ± 0.000	0.000 ± 0.000	0.038 ± 0.055	0.004 ± 0.007	0.060 ± 0.078	0.061 ± 0.090	0.061 ± 0.054	0.099 ± 0.139	0.095 ± 0.060
Ste-isoLCA	0.000 ± 0.000	0.001 ± 0.001	0.088 ± 0.104	0.007 ± 0.010	0.097 ± 0.075	0.078 ± 0.082	0.104 ± 0.078	0.205 ± 0.220	0.086 ± 0.047
Ole-isoLCA	0.000 ± 0.001	0.001 ± 0.001	0.052 ± 0.060	0.016 ± 0.024	0.123 ± 0.102	0.186 ± 0.248	0.110 ± 0.048	0.254 ± 0.343	0.129 ± 0.089
Lin-isoLCA	0.000 ± 0.000	0.000 ± 0.000	0.023 ± 0.027	0.007 ± 0.010	0.053 ± 0.048	0.080 ± 0.084	0.041 ± 0.024	0.079 ± 0.092	0.039 ± 0.030

Abbreviations of each FA-conjugated isoBA are shown in Fig. 1.

FA, fatty acid; SD, standard deviation; Unit: μmol/g.

**Fig. 6:**
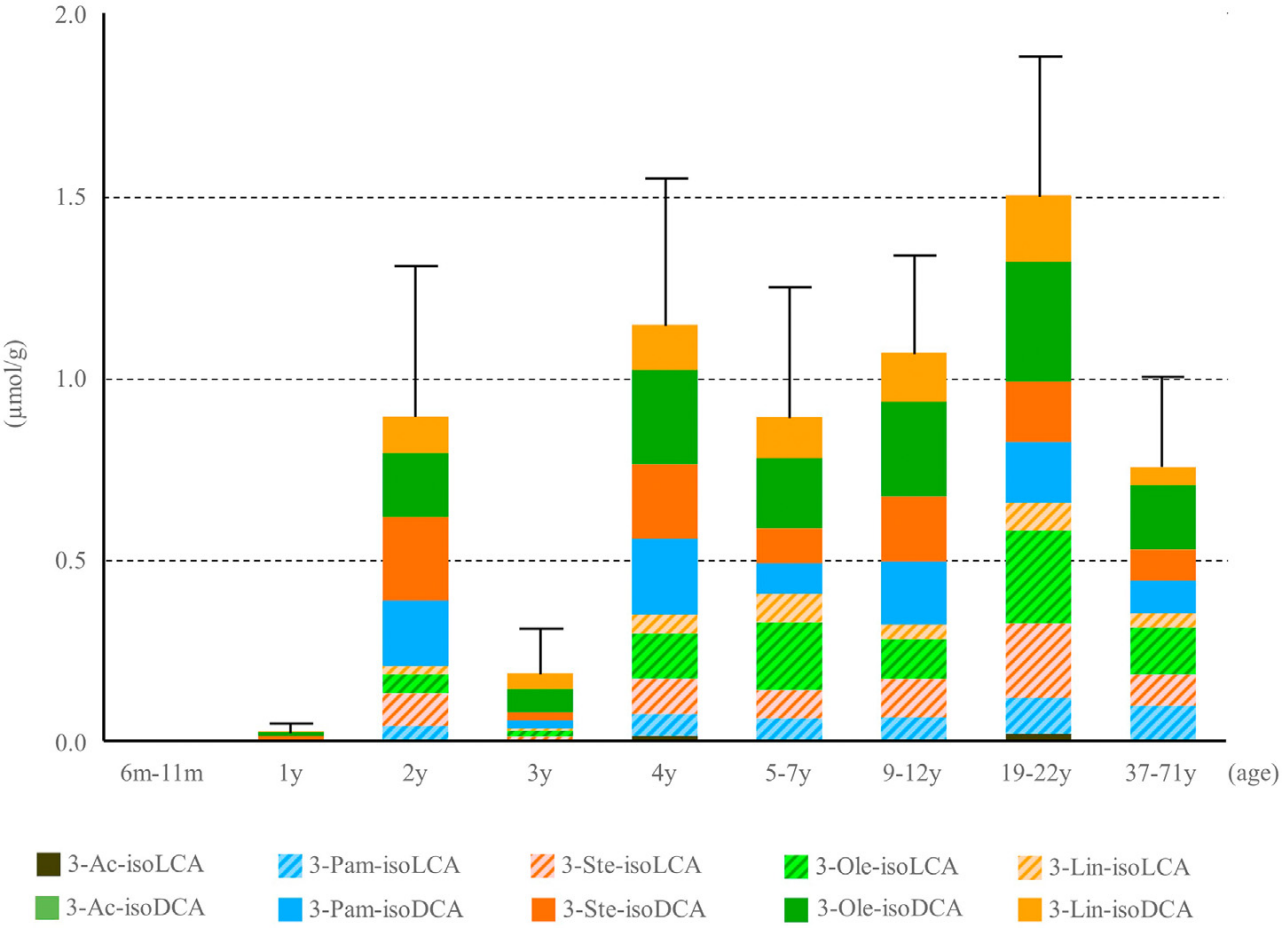
Comparison of fecal FA-isoBA concentrations by age group. Error bars represent SE of total FA-isoBA concentrations. Abbreviations of each FA-conjugated isoBA are shown in Fig. 1. BA, bile acid; FA, fatty acid.

The four examined FA acyl conjugates of isoDCA, Pam-isoDCA, Ste-isoDCA, Ole-isoDCA, and Lin-isoDCA were present in essentially equal amounts regardless of age group. Similarly, Pam-isoLCA, Ste-isoLCA, Ole-isoLCA, and Lin-isoLCA were present in essentially equal amounts in all age groups (Fig. 6). The data in Table 6 compares fecal concentrations of FA (Method A and B) and FA-isoBA. Interestingly, saturated FAs (Pam and Ste) are dominant over unsaturated FAs (Ole and Lin) in most age group. These observations imply that the esterification to isoBAs occurs more preferably in the unsaturated FAs. In addition to long-chain FA conjugates, small amounts of acetyl esters (3β-acetyl isoLCA) were found in the feces of some age groups. Since the recovery rates for Ac-isoBAs using our method were insufficient (Table 5), the true quantity of Ac-isoBAs may be higher than these values. The extraction method will need to be optimized for accurate quantification.

**Table 6:** Fecal fatty acid and FA-isoBA concentrations by age groups

	6–11 months (N=6)	1 year (N=6)	2 years (N=7)	3 years (N=7)	4 years (N=7)	5–7 years (N=7)	9–12 years (N=6)	19–22 years (N=14)	37–71 years (N=4)
	Mean ± SD	Mean ± SD	Mean ± SD	Mean ± SD	Mean ± SD	Mean ± SD	Mean ± SD	Mean ± SD	Mean ± SD
FA Method A									
Pam	63.70 ± 97.03	12.82 ± 6.42	23.56 ± 22.55	70.15 ± 80.36	46.54 ± 51.71	35.24 ± 22.81	55.13 ± 36.85	30.66 ± 32.71	17.03 ± 7.90
Ste	34.01 ± 27.20	7.74 ± 5.54	14.39 ± 19.17	31.43 ± 38.49	29.71 ± 24.48	24.04 ± 13.66	31.48 ± 17.21	24.13 ± 27.98	5.73 ± 2.10
Ole	5.73 ± 4.32	6.08 ± 5.01	4.98 ± 2.83	10.30 ± 9.86	8.75 ± 5.05	10.01 ± 6.72	10.41 ± 8.83	7.64 ± 6.66	8.97 ± 6.43
Lin	2.49 ± 4.00	3.15 ± 2.63	2.88 ± 2.78	8.00 ± 9.70	4.87 ± 3.03	5.70 ± 5.59	1.87 ± 1.28	4.01 ± 6.60	15.83 ± 21.13
FA Method B									
Pam	71.25 ± 102.54	19.85 ± 15.33	29.72 ± 27.08	112.71 ± 98.78	59.92 ± 57.00	50.36 ± 23.33	67.20 ± 43.67	40.08 ± 40.49	34.51 ± 34.60
Ste	37.92 ± 27.57	10.11 ± 9.26	16.63 ± 21.62	51.37 ± 42.40	36.86 ± 27.23	41.45 ± 19.39	36.71 ± 18.08	31.09 ± 34.75	13.75 ± 13.33
Ole	7.52 ± 5.58	14.98 ± 17.57	9.33 ± 7.44	35.82 ± 60.35	18.77 ± 11.36	10.46 ± 4.19	15.52 ± 13.27	14.02 ± 10.28	24.99 ± 36.84
Lin	2.28 ± 2.64	12.60 ± 23.23	7.12 ± 6.14	39.95 ± 85.11	13.16 ± 11.84	6.32 ± 5.91	4.17 ± 3.48	6.92 ± 9.24	23.38 ± 37.29
FA-isoBAs									
Pam-isoBAs	0.00 ± 0.00	0.00 ± 0.00	0.22 ± 0.26	0.03 ± 0.06	0.27 ± 0.35	0.15 ± 0.16	0.24 ± 0.19	0.27 ± 0.32	0.19 ± 0.11
Ste-isoBAs	0.00 ± 0.00	0.02 ± 0.03	0.32 ± 0.43	0.03 ± 0.05	0.30 ± 0.28	0.17 ± 0.14	0.28 ± 0.22	0.37 ± 0.36	0.17 ± 0.06
Ole-isoBAs	0.00 ± 0.00	0.01 ± 0.02	0.23 ± 0.23	0.08 ± 0.13	0.38 ± 0.28	0.38 ± 0.38	0.37 ± 0.15	0.58 ± 0.56	0.31 ± 0.14
Lin-isoBAs	0.00 ± 0.00	0.00 ± 0.00	0.12 ± 0.12	0.05 ± 0.08	0.18 ± 0.14	0.19 ± 0.17	0.18 ± 0.11	0.26 ± 0.23	0.09 ± 0.05

Abbreviations of each fatty acid-conjugated isobile acid are shown in Fig. 1.

FA, fatty acid; SD, standard deviation; Unit: μmol/g.

### FA-isoBAs in bile, serum, and urine

FA-isoBAs were undetectable in serum (N = 5), urine (N = 5), and gallbladder bile (N = 5) (data not shown).

## Discussion

Saponifiable BAs in human feces have been recognized since the incipient period of modern BA research (34). In an early study, Norman demonstrated that the administration of [^14^C]-CA results in the excretion of [^14^C]-3β,12α-dihydroxycholanic acid (isoDCA) in feces, of which a large portion is in the saponifiable form (30). Several saponifiable BAs, including ethyl esters of LCA and isoLCA (35), long-chain FA-conjugated isoLCA (16), and poly-DCA (28) have been reported. These saponifiable BAs have been estimated to constitute up to 25–30% of total fecal BAs (36). Despite being a major component of fecal BAs, saponifiable BAs have often been ignored in measurements, presumably due to need for tedious sample preparation or the unavailability of authenticated standards. Therefore, their biosynthesis, metabolism, and role in host physiology remain poorly understood.

Among the many fecal BAs with different structures, 3β-hydroxylated BAs (isoBAs) such as isoCDCA, isoUDCA, isoDCA, isoLCA, and 12β-hydroxy (or 12-oxo)-isoLCA are predominantly present as saponifiable forms. Long-chain FAs, Pam, Ste, Ole, and Lin were identified as major FA moieties linked to these isoBAs. In particular, FA-conjugated isoDCA and isoLCA accounted for 20.2% of total saponifiable BAs. Kelsey *et al.* demonstrated that incubation of [^14^C]-LCA-3S in a fecal culture yields [^14^C]-3β-palmityl, palmitoleyl, stearyl, and oleyl esters of isoLCA (16), which agrees with our current findings. Of note, the precursor ion scan spectra indicated the possible presence of myristic acid (C_14_H_28_O_2_)- and palmitoleic acid (C_16_H_30_O_2_)-linked isoDCA and isoLCA (Fig. 4). However, we did not quantitate the myristoyl and palmitoyl isoBAs, because their concentrations were much smaller than those of the other four major FA acyl isoBAs. We additionally found small amounts of acetic acid conjugates of isoLCA. Interestingly, it appears that FA acyl isoBAs were preferably linked with monohydroxy and dihydroxy BAs (i.e., isoLCA and isoDCA). There was much less presence of FA-linked trihydroxy isoBAs (i.e., isoCA and isoUCA). It appears there were no FA-linked 3α-hydroxy BAs (i.e., DCA and LCA). Both studies by Norman (30) and Kelsey (16) also preferentially identified monohydroxy and dihydroxy isoBAs as FA-linked BAs, which agreed with our observations.

Traditionally, the saponifiable fraction of fecal BAs has been indirectly estimated after alkaline treatment. In our previous study, we used 0.1 M NaOH in 50% aqueous isopropanol and incubated the fecal extract at 60°C for 2 h. This relatively mild condition quantitatively cleaved ethyl lithocholate and ethyl lithocholyl stearate standards (22). However, in the present study, we found there were more BAs that can be hydrolyzed when fecal extracts were treated with 1 M methanolic KOH at 90°C for 1 h. This finding suggests that more severe conditions were needed for the complete hydrolysis of fecal BA esters. Over time, various conditions from milder (i.e., 0.7 M KOH in methanol at room temperature overnight) (37) to more severe (i.e., 1 M NaOH in 90% ethanol at 110°C for 18 h) (28) have been attempted for the hydrolysis of fecal BAs. Using a milder condition such as in our previous study (22) could possibly lead to underestimation of saponifiable BAs due to incomplete hydrolysis. Meanwhile, the use of strong alkaline and high temperature can cause epimerization of 3α-hydroxy groups to 3β-hydroxy groups (isoBAs). As a result, the overestimation of FA-isoBAs becomes a major concern. Also, this epimerization could cause underestimation of 3α-hydroxy BAs (i.e., DCA and LCA).

The quantitation of FA-isoBAs in fecal specimens was based on retention time, exact mass, and MS/MS fragmentation patterns and comparisons with authenticated standards. The negative MS/MS spectra of FA-linked 3α-hydroxy BA commonly gave [BA-H]^−^ and [BA-H_2_O-H]^−^, whereas FA 3β-hydroxy-linked BAs (FA-isoBAs) gave [BA-H_2_O-H]^−^ as the base fragment ions. Therefore, we used [isoLCA-H_2_O-H]^−^ for quantification of FA-isoLCAs. Meanwhile, [isoDCA-H_2_O-H]^−^ was not suitable as the quantitation ion for most FA-isoDCAs due to the appearance of interferent peaks at close retention times in the analytes. Therefore, we selected [FA-H]^−^ for the quantitation of FA-isoDCAs. We assessed the matrix effects as well as extraction efficiency of the FA-isoBAs according to the method of Matuszewski (33). When spiked Bligh-Dyer extract was used for the evaluation, the recovery rate of long-chain FA-isoBAs was 92.1–147.6%. The total recovery rate from the spiked dry stool was 73.2–102.8% depending on the long-chain FA-isoBAs. Meanwhile, the present method had poor recovery rates for Ac-isoBAs. We found Ac-isoBAs can be recovered 109–161% when authentic Ac-isoBAs were spiked to the Bligh-Dyer extract (data not shown). It is possible that they were hydrolyzed during the fecal extraction in 0.1 M NaOH solution. If this is the case, the recovery rate should be improved when alcohol based solution is used for the fecal extraction. For more accurate quantitation of both long-chain FA-isoBA and Ac-isoBAs, isotope-labeled ISs need to be added for all analytes.

Most likely, gut microbes are responsible for FA-isoBA production, since no detectable levels of any FA-isoBAs were found in bile, serum, and urine. Traditionally, only four distinct reactions including deconjugation, dehydroxylation, oxidation, and epimerization have been recognized as major microbial reactions for BAs. However, there is enough evidence of microbial “re-conjugation” of BAs, including the early studies by Norman and Kelsey *et al.* (16, 30). Sugai *et al.* also demonstrated the esterification of C_12_-C_16_ FAs with 3α-hydroxy BAs using *Candida antarctica* lipase (38). Unfortunately, this group did not test the esterification with 3β-hydroxy BAs by this enzyme. More recently, C24-aminoacyl conjugation of CA with specific amino acids (i.e., phenylalanine, leucine, and tyrosine) has been shown by the gut microbiota, just like the aminoacyl conjugation (taurine, glycine) occurs in the host liver (39). The bacterium *Enterocloster bolteae* (*Clostridium bolteae*) has been identified as a mediator of this acyl conjugation. Thus, reconjugation is a common microbial transformation of BAs, and therefore it should be recognized as a fifth distinct microbial transformation. FA acylation to isoBA is a representative example of such a microbial “re-conjugation”.

Most secondary BA-transforming bacteria start colonization and proliferation in the gut soon after baby-food supplementation begins (40). DCA and LCA are epimerized to isoDCA and isoLCA by *Clostridium perfringens* (41). Sulfatases produced by *Clostridium* and *Pseudomonas aeruginosa* catalyze the hydrolysis of LCA-3S (42, 43). Our analysis showed that FA-isoBA was undetectable in post-neonate (6–11 months) feces. It first appeared in 1-year-olds, and the amount was significantly elevated in 2-year-olds. These results clearly suggest that the development of FA-isoBA-conjugating bacteria is closely correlated with the onset of weaning. Once the microbiome is established, FA-isoBA is constantly secreted throughout life. Of note, we ran the product ion mass scan analysis for three fecal specimens from infants (6–12 months old, all males) in whose stools were detected isoCDCA, but we did not detect DCA or LCA. The parental ion scan data based on a product ion of *m/z* 373 [isoCDCA-H]^−^ indicated that long-chain FA-isoCDCA esters were possibly present in these specimens (Fig. 4, Peaks 17 and 18). These data indicated that bacterial colonization resulting in isomerization of the 3β-hydroxy group and FA esterification may possibly begin earlier than weaning. Analysis of more samples is needed to confirm this observation.

The role of FA-isoBAs in host physiology remains unclear. Gilat *et al.* demonstrated synthetic long-chain FA (C-6 to C-22)-linked CA prevented cholesterol crystallization in human bile (44). These compounds were absorbed from the intestine, secreted into the bile, and prevented cholesterol gall stone formation. It has also been reported that long-chain FA conjugation to the 3α-position of BA weakens antibacterial activity (38). FA conjugation to BA may play more important roles in maintaining the host intestinal environment or regulating immunity. Some possible mechanisms have been shown: short-chain FA-conjugated LCA binds to vitamin D receptors (45, 46, 47), inhibiting the proliferation of human monoblastic leukemia cells and inducing their monocytic differentiation. Ac-LCA binds to vitamin D receptor and activates the receptor with 30 times the potency of LCA (47). Recently, isoalloLCA was shown to reduce T helper cells expressing interleukin-17a (Th17 cells) and increase regulatory T cell differentiation in the intestinal lamina propria. Long-chain FA conjugation may partially contribute to a mechanism by which BA metabolites control host immune responses by directly modulating the Th17 and regulatory T cell balance (48).

**Fig. 7:**
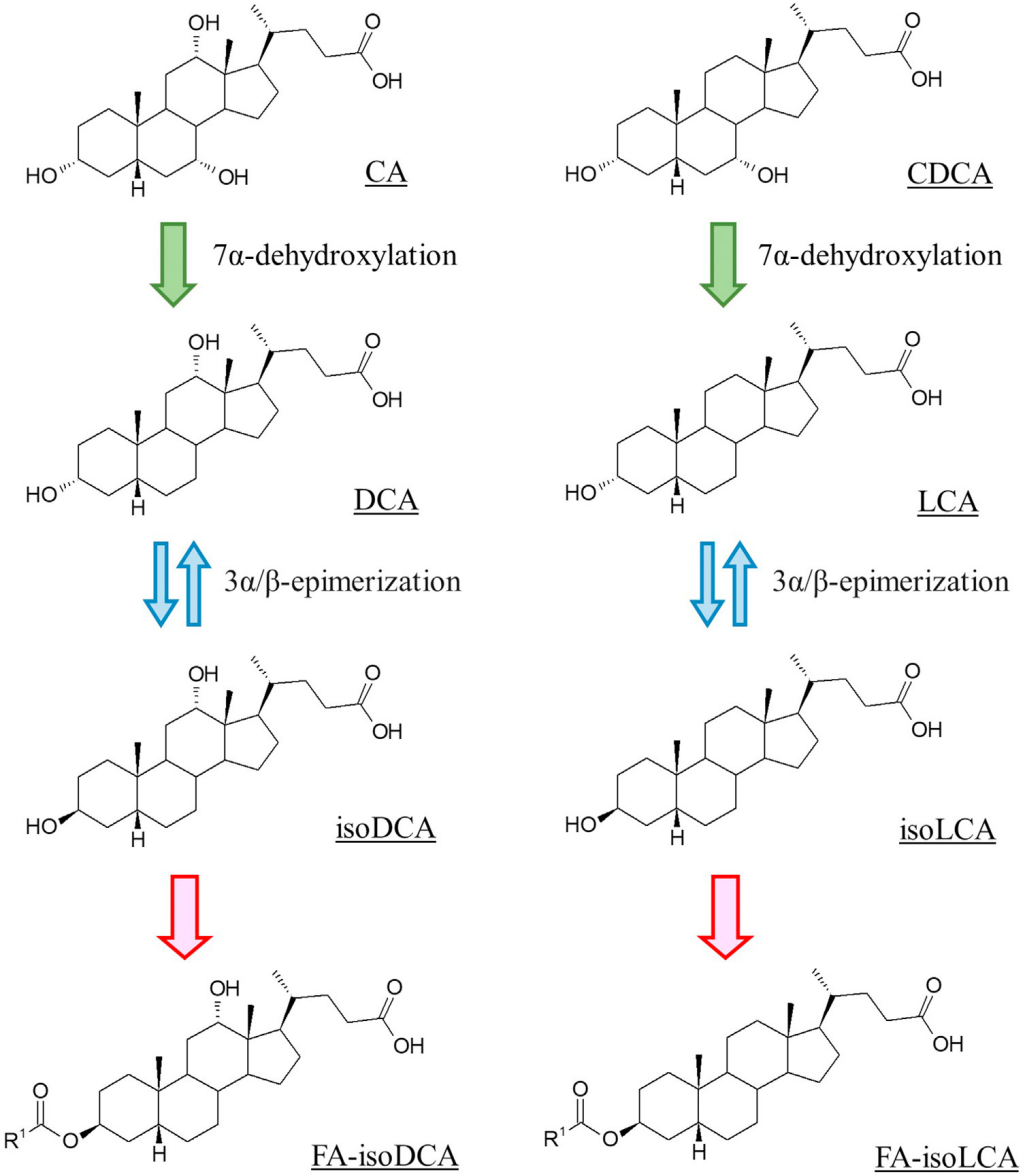
Potential microbial conversion of CA and CDCA to FA-isoDCA and FA-isoLCA. isoDCA, isodeoxycholic acid; isoLCA, isolithocholic acid; CA, cholic acid; CDCA, chenodeoxycholic acid; FA, fatty acid.

Additionally, microbial long-chain FA conjugation might work as a guest-host mechanism to reduce cytotoxic BAs. The epimerization of 3α-hydroxy groups in DCA and LCA to form isoDCA and isoLCA weakens their toxicity (12, 49). Since the conversion between two epimers (3β-OH ↔ 3α-OH) is reversible, binding of FA fixes the orientation of hydroxyl groups and prevents isoBAs from returning to toxic 3α-hydroxy BAs (Fig. 7). In this context, among 52 “healthy” fecal specimens examined (2 years or older), there were seven cases in which the concentrations of FA-isoDCA or FA-isoLCA were extremely low (below the LOQ of the system), and among four of these cases, FA-isoDCA and FA-isoLCA were not detected at all. Interestingly, three of the FA-isoBAs undetected samples had normal levels of isoDCA and isoLCA. These data suggests that these individuals lacked FA esterification bacteria. Plasma biochemical data (lipid, cholesterol, aspartate aminotransferase, alanine aminotransferase, glucose, insulin, etc.) of these individuals were normal.

In summary, we have developed a robust analytical method for fecal BA with a series of synthetic FA-BAs. We comprehensively analyzed fecal FA-BA in healthy specimens across a broad age range and identified long-chain FA (C16 and C18) acyl-conjugated isoBAs as a major saponifiable fraction of fecal BAs. A small amount of short-chain FAs, such as acetic acid conjugates, was also found. Fecal FA-isoBAs were present in most people over 2 years of age. It appears age, sex, or fecal FA concentrations were not correlated to the fecal FA-isoBA concentrations. The role of FA-isoBAs in host physiology, their contribution to gastroenteric or hepatic diseases, and the identification of gut bacteria involved in the production of FA-isoBAs are currently under investigation in our laboratories.

## Data availability

All data are contained within this article. The raw data will be shared upon request: Contact Hiroshi Nittono (Junshin Clinic Bile Acid Institute, Email: bile-res@eco.ocn.ne.jp).

## Supplemental data

This article contains supplemental data.

## Conflict of interest

The authors declare that they have no conflicts of interest with the contents of this article.
