# Supplementary material for: Characterization of long-chain fatty acid-linked bile acids: a major conjugation form of 3β-hydroxy bile acids in feces
Source: J Lipid Res. 2022 Sep 9;63(10):100275. doi: 10.1016/j.jlr.2022.100275 (PMC9587409; doi:10.1016/j.jlr.2022.100275)
Supplement: Supplemental Data [file mmc1.pdf]

Supplemental Text S1: Chemical synthesis of 3β-fatty acid-conjugated bile acids

Synthesis of methyl 3β-hydroxylithocholate (Me-isoLCA) and methyl 3β-hydroxydeoxycholate (Me-isoDCA)

To a solution of methyl lithocholate (1.0 mmol) in tetrahydrofuran (THF) (5 ml), triphenylphosphine (2.1 mmol), 98% formic acid (2.1 mmol), and dimethylazocarboxylate (2.1 mmol) were added. The mixture was stirred at room temperature (r.t.) for 30 min. After the solvent was evaporated under reduced pressure, the residue was redissolved in *n*-hexane and applied on a column of silica gel (*n*-hexane/ethyl acetate (AcOEt) = 10:1 v/v). The fraction containing Me-isoLCA was monitored by thin-layer chromatography (TLC) and combined. After evaporation of the solvent, the obtained colorless solids (*ca.* 0.83 mmol) were treated with 0.5 M methanolic KOH for 10 min. The solution was neutralized with 2 M HCl. Me-isoLCA was extracted with AcOEt. The organic phase was successively washed with *sat.* $NaHCO_3$, brine, and then dried over anhydrous Na_2SO_4. After evaporation of the solvent under reduced pressure, the desired Me-isoLCA was obtained as a dried colorless solid (0.79 mmol). ^{1}H-NMR ($CDCl_3$, 500 MHz): δ: 0.64 (3H, s, 18-CH_3), 0.90 (3H, d, J=6.2 Hz, 21-CH_3), 0.95 (3H, s, 19-CH_3), 3.65 (3H, s, $COOCH_3$), 4.10 (1H, bs, 3α-H), 0.87 (t, 3H, J=6.9 Hz, -CH_3).

Me-isoDCA was synthesized from methyl deoxycholate (1.0 mmol) in the same manner, except for changing the reagent quantity: THF (5 ml), triphenylphosphine (2.5 mmol), 98% formic acid (2.5 mmol), and dimethylazocarboxylate (2.5 mmol). For silica gel column chromatography, benzene/AcOEt, 10:1, v/v was used for the eluent. Me-isoDCA was obtained as a dried colorless amorphous solid (0.79 mmol). ^{1}H-NMR ($CDCl_3$, 500 MHz): δ: 0.68 (3H, s, 18-CH_3), 0.94 (3H, s, 19-CH_3), 0.90 (3H, d, J=6.3 Hz, 21-CH_3), 3.66 (3H, s, $COOCH_3$), 3.98 (1H, bs, 12β-H), 4.10 (1H, bs, 3α-H).

Synthesis of 3β-acetyl isolithocholic acid (Ac-isoLCA) and 3β-acetyl isodeoxycholic acid (Ac-isoDCA)

To a solution of Me-isoLCA (0.3 mmol) in benzotrifluoride (3 ml), anhydrous acetic acid (0.4 mmol), triethylamine (0.4 mmol), and dimethylaminopyridine (0.1 mmol) were added. The mixture was stirred at r.t. for 1 h. After evaporation of the solvent, the residue was redissolved in benzene and applied on a column of silica gel (benzene/AcOEt = 40:1 v/v). The effluents were fractionated and evaporated to dryness under reduced pressure. The desired methyl isolithocholate 3β-yl acetate was collected as a crude colorless solid (0.28 mmol), which was successively subjected to selective de-methylation without further purification; the crude solid was redissolved in dry pyridine (4 ml). To the solution, LiI (1.2 mmol) was added, and the mixture was refluxed for 72 h. After the addition of AcOEt (*ca*. 100 ml), the organic phase was washed with 2 M HCl, then brine, and then dried over anhydrous $MgSO_4$. The solvent was evaporated, and the residue was redissolved in benzene (*ca.* 5 ml) and applied on a column of silica gel (benzene/AcOEt = 10:1 v/v). The effluent was fractionated and monitored by TLC. The combined effluents were dried under reduced pressure. The desired Ac-isoLCA was collected as a colorless amorphous solid (0.24 mmol). ^{1}H-NMR ($CDCl_3$, 500 MHz), δ: 0.65 (3H, s, 18-CH_3), 0.92 (3H, d, J=6.2 Hz, 21-CH_3), 0.96 (3H, s, 19-CH_3), 2.04 (3H, s, OCH_3), 5.07 (1H, bs, 3α-H).

Ac-isoDCA was prepared from Me iso-DCA (0.25 mmol) in essentially the same manner. The quantities of reagents were as follows: benzotrifluoride (3 ml), anhydrous acetic acid (0.33 mmol), triethylamine (0.33 mmol), dimethylaminopyridine (0.1 mmol). Benzene/AcOEt, 5:1, v/v was used for the purification of methyl isodeoxycholate 3β-yl acetate, which was obtained as a colorless solid (0.13 mmol) (II, R_1: OH). This intermediate compound was similarly demethylated. The desired Ac-isoDCA was obtained as a dried colorless solid (0.09 mmol). ^{1}H-NMR ($CDCl_3$, 500 MHz), δ: 0.68 (3H, s, 18-CH_3), 0.95 (3H, s, 19-CH_3), 0.98 (3H, d, J=6.2 Hz, 21-CH_3), 2.04 (3H, s, OCH_3), 3.99 (1H, bs, 12β-H), 5.07 (1H, bs, 3α-H).

Synthesis of long-chain fatty acid conjugates of isoLCA and isoDCA

To a solution of Me-isoLCA or Me-isoDCA (0.22 mmol) in THF (4 ml for Me-isoLCA; 3 ml for Me-isoDCA), fatty acid (palmitic acid, stearic acid, oleic acid, or linoleic acid; 0.4–0.6 mmol), *N,N′*-diisopropylcarbodiimide (0.5–0.8 mmol), and dimethylaminopyridine (0.8 mmol) were added. The mixture was stirred at r.t. for 12 h. After evaporation of the solvent, the residue was redissolved in benzene and applied on a column of silica gel (benzene/AcOEt, 40:1 v/v). The solvent in the combined fraction was evaporated under reduced pressure. The obtained 3β-fatty acid conjugate of methyl isocholanoate (colorless solid) was successively subjected to selective demethylation in the same manner as described for the synthesis of Ac-isoLCA. After being purified by silica gel column chromatography, the desired 3β-fatty acid conjugates of isoLCA and isoDCA were obtained as colorless solids as follows:

3β-Hexadecanoyloxy-5β-cholanoic acid

(3β-Palmitoyl isolithocholic acid: Pam-isoLCA)

Yield, 0.25 mmol, ^{1}H-NMR: δ: 0.64 (3H, s, 18-CH_3), 0.87 (3H, t, J=6.9 Hz, -CH_3), 0.92 (3H, d, J=6.2 Hz, 21-CH_3), 0.96 (3H, s, 19-CH_3), 1.25-1.28 (m, 26H, -CH_2-), 2.28 (2H, t, J=7.4 Hz, CO-CH_2-), 5.07 (1H, bs, 3α-H).

3β-Octadecanoyloxy-5β-cholanoic acid

(3β-Stearyl isolithocholic acid: Ste-isoLCA)

Yield, 0.16 mmol, ^{1}H-NMR: δ: 0.64 (3H, s, 18-CH_3), 0.87 (3H, t, J=6.9 Hz, -CH_3), 0.92 (3H, d, J=6.2 Hz, 21-CH_3), 0.96 (3H, s, 19-CH_3), 1.25-1.28 (m, 30H, -CH_2-), 2.28 (3H, t, J=7.4 Hz, CO-CH_2-), 5.07 (1H, bs, 3α-H).

3β-(9-Cis-octadecenoyloxy)-5β-cholanoic acid

(3β-Oleyl isolithocholic acid: Ole-isoLCA)

Yield, 0.17 mmol, ^{1}H-NMR: δ: 0.65 (3H, s, 18-CH3), 0.87 (3H, t, J=6.9 Hz, -CH_3), 0.92 (3H, d, J=6.2 Hz, 21-CH_3), 0.96 (3H, s, 19-CH_3), 1.25-1.28 (m, 22H, -CH_2-), 1.99 (4H, m, -CH_2-CH=CH-CH_2-), 2.28 (2H, t, J=7.4 Hz, CO-CH_2-), 5.07 (1H, bs, 3α-H), 5.33-5.34 (2H, m, -CH=CH-).

3β-(9-Cis,12-cis-octadecadienoyloxy)-5β-cholanoic acid

(3β-Linoleyl isolithocholic acid: Lin-isoLCA)

Yield, 0.13 mmol, ^{1}H-NMR: δ: 0.65 (3H, s, 18-CH_3), 0.88 (3H, t, J=6.9 Hz, -CH_3), 0.92 (3H, d, J=6.2 Hz, 21-CH_3), 0.96 (3H, s, 19-CH_3), 1.25-1.28 (m, 16H, -CH_2-), 2.03-2.05 (4H, m, -CH_2-CH=CH-CH_2-), 2.29 (2H, t, J=7.4 Hz, CO-CH_2-), 2.75-2.77 (2H, m, =CH-CH_2-CH=), 5.07 (1H, bs, 3α-H), 5.32-5.37 (4H, m, (-CH=CH-)×2).

3β-Hexadecanoyloxy-12α-hydroxy-5β-cholanoic acid

(3β-Palmitoyl isodeoxycholic acid: Pam-isoDCA)

Yield, 0.09 mmol, m.p., ^{1}H-NMR: δ: 0.68 (3H, s, 18-CH_3), 0.87 (3H, t, J=6.9 Hz, -CH_3), 0.94 (3H, s, 19-CH_3), 0.96 (3H, d, J=6.3 Hz, 21-CH_3), 1.25-1.28 (m, 26H, -CH_2-), 2.28 (3H, t, J=7.4 Hz, CO-CH_2-), 3.99 (1H, bs, 12β-H), 5.07 (1H, bs, 3α-H).

3β-Octadecanoyloxy-12α-hydroxy-5β-cholanoic acid

(3β-Stearyl isodeoxycholic acid: Ste-isoDCA)

Yield, 0.11 mmol, m.p., ^{1}H-NMR: δ: 0.68 (3H, s, 18-CH_3), 0.87 (3H, t, J=6.9 Hz, -CH_3), 0.95 (3H, s, 19-CH_3), 0.98 (3H, d, J=6.3 Hz, 21-CH_3), 1.25-1.28 (m, 30H, -CH_2-), 2.28 (3H, t, J=7.4 Hz, CO-CH_2-), 3.99 (1H, bs, 12β-H), 5.07 (1H, bs, 3α-H).

3β-(9-Cis-octadecenoyloxy)-12α-hydroxy-5β-cholanoic acid

(3β-Oleyl isodeoxycholic acid: Ole-isoDCA)

Yield, 0.12 mmol, ^{1}H-NMR: δ: 0.69 (3H, s, 18-CH_3), 0.87 (3H, t, J=6.9 Hz, -CH_3), 0.95 (3H, s, 19-CH_3), 0.98 (3H, d, J=6.2 Hz, 21-CH_3), 1.26-1.29 (m, 22H, -CH_2-), 2.00 (4H, m, -CH_2-CH=CH-CH_2-), 2.28 (2H, t, J=7.4 Hz, CO-CH_2-), 3.99 (1H, bs, 12β-H), 5.07 (1H, bs, 3α-H), 5.33-5.34 (2H, m, -CH=CH-).

3β-(9-Cis,12-cis-octadecadienoyloxy)-12α-hydroxy-5β-cholanoic acid

(3β-Linoyl isodeoxycholic acid: Lin-isoDCA)

Yield, 0.10 mmol, ^{1}H-NMR: δ: 0.68 (3H, s, 18-CH_3), 0.88 (3H, t, J=6.9 Hz, -CH_3), 0.95 (3H, s, 19-CH_3), 0.96 (3H, d, J=6.2 Hz, 21-CH_3), 1.28-1.35 (m, 16H, -CH_2-), 2.03-2.05 (4H, m, -CH_2-CH=CH-CH_2-), 2.29 (2H, t, J=7.4 Hz, CO-CH_2-), 2.75-2.77 (2H, m, =CH-CH_2-CH=), 3.99 (1H, bs, 12β-H), 5.07 (1H, bs, 3α-H), 5.32-5.37 (4H, m, (-CH=CH-)×2).
