# Supplementary material for: Characterization of long-chain fatty acid-linked bile acids: a major conjugation form of 3β-hydroxy bile acids in feces
Source: J Lipid Res. 2022 Sep 9;63(10):100275. doi: 10.1016/j.jlr.2022.100275 (PMC9587409; doi:10.1016/j.jlr.2022.100275)
Supplement: Supplemental Table 1 [file mmc2.pdf]

Supplemental Table S1 (Appendix)

Abbreviations and corresponding trivial names of the Fatty acid-bile acid esters, fatty acids, and bile acids used in this study.

Abbreviations	Trivial names	Structure Lipid Maps ID
Fatty acid-bile acid ester		
Ac-isoLCA	3β-acetoxy-5β-cholanoic acid	
Pam-isoLCA	3β-hexadecanoyloxy-5β-cholanoic acid	
Ste-isoLCA	3β-octadecanoyloxy-5β-cholanoic acid	
Ole-isoLCA	3β-(9-cis-octadecenoyloxy)-5β-cholanoic acid	
Lin-isoLCA	3β-(9-cis,12-cis-octadecadienoyloxy)-5β-cholanoic acid	
Ac-isoDCA	3β-acetoxy-12α-hydroxy-5β-cholanoic acid	
Pam-isoDCA	3β-hexadecanoyloxy-12α-hydroxy-5β-cholanoic acid	
Ste-isoDCA	3β-octadecanoyloxy-12α-hydroxy-5β-cholanoic acid	
Ole-isoDCA	3β-(9-cis-octadecenoyloxy)-12α-hydroxy-5β-cholanoic acid	
Lin-isoDCA	3β-(9-cis,12-cis-octadecadienoyloxy)-12α-hydroxy-5β-cholanoic acid	
Ac-LCA	3α-acetoxy-5β-cholanoic acid	
Ste-LCA	3α-octadecanoyloxy-5β-cholanoic acid	
Ste-DCA	3α-octadecanoyloxy-12α-hydroxy-5β-cholanoic acid	
Fatty acid		
Palmitic acid	hexadecanoic acid	LMFA01010001
Stearic acid	octadecanoic acid	LMFA01010018
Oleic acid	9-cis-Octadecenoic acid	LMFA01030002
Linoleic acid	9-cis,12-cis-Octadecadienoic acid	LMFA01030120
Bile acid		
CA	cholic acid	LMST04010001
alloCA	3α,7α,12α-trihydroxy-5α-cholanoic acid	LMST04010092
isoCA	3β,7α,12α-trihydroxy-5β-cholanoic acid	LMST04010086
3-oxo-CA	7α,12α-dihydroxy-3-oxo-5β-cholanic acid	LMST04010443
7-oxo-DCA	3α,12α-dihydroxy-7-oxo-5β-cholanoic acid	LMST04010184
12-oxo-CDCA	3α,7α-dihydroxy-12-oxo-5β-cholanic acid	LMST04010180
UCA	3α,7β,12α-trihydroxy-5β-cholanoic acid	LMST04010088
isoUCA	3β,7β,12α-trihydroxy-5β-cholanoic acid	LMST04010090
HCA	3α,6α,7α-trihydroxy-5β-cholanoic acid	LMST04010064
CDCA	chenodeoxycholic acid	LMST04010032
alloCDCA	3α,7α-dihydroxy-5α-cholanoic acid	LMST04010036
isoCDCA	3β,7α-dihydroxy-5β-cholanoic acid	LMST04010034

3-oxo-CDCA	7α-hydroxy-3-oxo-5β-cholanic acid	LMST04010161
3-oxo-alloCDCA	7α-hydroxy-3-oxo-5α-cholanic acid	LMST04010163
7-oxo-LCA	3α-hydroxy-7-oxo-5β-cholanic acid	LMST04010150
UDCA	Ursodeoxycholic acid	LMST04010033
isoUDCA	3α,7β-dihydroxy-5β-cholanoic acid	LMST04010035
3-oxo-UDCA	7β-hydroxy-3-oxo-5β-cholanic acid	LMST04010162
DCA	deoxycholic acid	LMST04010040
alloDCA	3α,12α-dihydroxy-5α-cholanoic acid	LMST04010044
isoDCA	3β,12α-dihydroxy-5β-cholanoic acid	LMST04010042
isoalloDCA	3β,12α-dihydroxy-5α-cholanoic acid	LMST04010046
3-oxo-DCA	12α-hydroxy-3-oxo-5β-cholanic acid	LMST04010168
3-oxo-alloDCA	12α-hydroxy-3-oxo-5α-cholanic acid	LMST04010169
12β-OH-LCA	3α,12β-dihydroxy-5β-cholanoic acid	LMST04010045
12β-OH-isoLCA	3β,12β-dihydroxy-5β-cholanoic acid	LMST04010047
12-oxo-LCA	3α-hydroxy-12-oxo-5β-cholanic acid	LMST04010155
12-oxo-isoLCA	3β-hydroxy-12-oxo-5β-cholanic acid	LMST04010156
LCA	lithocholic acid	LMST04010003
alloLCA	3α-hydroxy-5α-cholanoic acid	LMST04010005
isoLCA	3β-hydroxy-5β-cholanoic acid	LMST04010004
isoalloLCA	3β-hydroxy-5α-cholanoic acid	LMST04010006
3-oxo-LCA	3-oxo-5β-cholanic acid	LMST04010127
3-oxo-alloLCA	3-oxo-5α-cholanic acid	LMST04010128
HDCA	3α,6α-dihydroxy-5β-cholanoic acid	LMST04010024
MDCA	3α,6β-dihydroxy-5β-cholanoic acid	LMST04010025
CA-Δ^{4}-3-one	7α,12α-dihydroxy-3-oxo-4-cholenoic acid	LMST04030114
DCA-$\Delta^{4,6}$-3-one	12α-hydroxy-3-oxo-4,6-choladienoic acid	LMST04010245
CDCA-Δ^{4}-3-one	7α-hydroxy-3-oxo-4-cholenoic acid	LMST04030123
LCA-$\Delta^{4,6}$-3-one	3-oxo-4,6-choladienoic acid	LMST04010235
DCA-Δ^{4}-3-one	12α-hydroxy-3-oxo-4-cholenoic acid	LMST04010198
LCA-Δ^{4}-3-one	3-oxo-4-cholenoic acid	LMST04010233
nor-CA	3,7,12-trihydroxy-24-nor-5β-cholan-23-oic acid	LMST04060019
GCA	glycocholic acid	LMST05030001
TCA	taurocholic acid	LMST05040001
CA-3S	cholic acid 3-sulfate	
GCA-3S	glycocholic acid 3-sulfate	
TCA-3S	taurocholic acid 3-sulfate	LMST05020031
GCDCA	glycochenodeoxycholic acid	LMST05030008

TCDCA	taurochenodeoxycholic acid	LMST05040005
CDCA-3S	chenodeoxycholic acid 3-sulfate	LMST05020024
GCDCA-3S	glycochenodeoxycholic acid 3-sulfate	
TCDCA-3S	taurochenodeoxycholic acid 3-sulfate	LMST05020029
GUDCA	glycoursodeoxycholic acid	LMST05030016
TUDCA	tauroursodeoxycholic acid	LMST05040015
UDCA-3S	ursodeoxycholic acid 3-sulfate	LMST05020033
GUDCA-3S	glycoursodeoxycholic acid 3-sulfate	LMST05030012
TUDCA-3S	tauroursodeoxycholic acid 3-sulfate	
DCA-3S	deoxycholic acid 3-sulfate	LMST05020038
GDCA	glycodeoxycholic acid	LMST05030006
TDCA	taurodeoxycholic acid	LMST05040013
GDCA-3S	glycodeoxycholic acid 3-sulfate	
TDCA-3S	taurodeoxycholic acid 3-sulfate	
GLCA	glycolithocholic acid	LMST05030009
TLCA	taurolithocholic acid	LMST05040003
LCA-3S	lithocholic acid 3-sulfate	LMST05020015
GLCA-3S	glycolithocholic acid 3-sulfate	LMST05030004
TLCA-3S	taurolithocholic acid 3-sulfate	LMST05020003
GHCA	glycohyocholic acid	
THCA	taurohyocholic acid	LMST05040010
GCA-1β-ol	glyco 1β,3α,7α,12α-tetrahydroxy-5β-cholanoic acid (glyco1β-hydroxy cholic acid)	
TCA-1β-ol	tauro 1β,3α,7α,12α-tetrahydroxy-5β-cholanoic acid (tauro1β-hydroxy cholic acid)	
CA-1β-ol	1β,3α,7α,12α-tetrahydroxy-5β-cholanoic acid (1β-hydroxy cholic acid)	LMST04010114
GCA-6α-ol	glyco 3α,6α,7α,12α-tetrahydroxy-5β-cholanoic acid (glyco 6α-hydroxy cholic acid)	
TCA-6α-ol	tauro 3α,6α,7α,12α-tetrahydroxy-5β-cholanoic acid (tauro 6α-hydroxy cholic acid)	
CA-6α-ol	3α,6α,7α,12α-tetrahydroxy-5β-cholanoic acid (6α-hydroxy cholic acid)	LMST04010118
CDCA-1β-ol	1β,3α,7α-trihydroxy-5β-cholanoic acid (1β-hydroxy chenodeoxycholic acid)	LMST04010058
GCA-Δ^4-3-one	glyco 7α,12α-dihydroxy-3-oxo-4-cholenoic acid	
TCA-Δ^4-3-one	tauro 7α,12α-dihydroxy-3-oxo-4-cholenoic acid	
GCDCA-Δ^4-3-one	glyco 7α-hydroxy-3-oxo-4-cholenoic acid	LMST05030020

TCDCA-Δ^4-3-one	tauro 7α-hydroxy-3-oxo-4-cholenoic acid	
Δ^5-3β-ol	3β-hydroxy-5-cholenoic acid	LMST04010201
GΔ^5-3β-ol	glyco 3β-hydroxy-5-cholenoic acid	
TΔ^5-3β-ol	tauro 3β-hydroxy-5-cholenoic acid	
Δ^5-3β-ol-3S	3β-hydroxy-5-cholenoic acid 3-sulfate	
GΔ^5-3β-ol-3S	glyco 3β-hydroxy-5-cholenoic acid 3-sulfate	
TΔ^5-7α-ol-3S	tauro 3β-hydroxy-5-cholenooic acid 3-sulfate	
Δ^5-3β,7α-diol	3β,7α-dihydroxy-5-cholenoic acid	LMST04010217
GΔ^5-3β,7α-diol	glyco 3β,7α-dihydroxy-5-cholenoic acid	
TΔ^5-3β,7α-diol	tauro 3β,7α-dihydroxy-5-cholenoic acid	
Δ^5-3β,7α-diol-3S	3β,7α-dihydroxy-5-cholen-24-oic acid 3-sulfate	
GΔ^5-3β,7α-diol-3S	glyco 3β,7α-dihydroxy-5-cholenoic acid 3-sulfate	
TΔ^5-3β,7α-diol-3S	tauro 3β,7α-dihydroxy-5-cholenoic acid 3-sulfate	
Δ^5-3β,7α,12α-triol	3β,7α,12α-trihydroxy-5-cholenoic acid	LMST04010227
GΔ^5-3β,7α12α-triol	glyco 3β,7α,12α-trihydroxy-5-cholenoic acid	
TΔ^5-3β,7α12α-triol	tauro 3β,7α,12α-trihydroxy-5-cholenoic acid	
Δ^5-3β,7α,12α-triol-3S	3β,7α,12α-trihydroxy-5-cholen-24-oic acid 3-sulfate	
GΔ^5-3β,7α12α-triol-3S	glyco 3β,7α,12α-trihydroxy-5-cholenoic acid 3-sulfate	
TΔ^5-3β,7α12α-triol-3S	tauro 3β,7α,12α-trihydroxy-5-cholenoic acid 3-sulfate	
