# Supplementary material for: Characterization of long-chain fatty acid-linked bile acids: a major conjugation form of 3β-hydroxy bile acids in feces
Source: J Lipid Res. 2022 Sep 9;63(10):100275. doi: 10.1016/j.jlr.2022.100275 (PMC9587409; doi:10.1016/j.jlr.2022.100275)
Supplement: Supplemental Tables 2–5 [file mmc3.pdf]

Supplemental table S2. LC/MS parameters for the BA quantitation in Condition II

Bile acid	RT (min)	Precursor ion (m/z)	Product ion (m/z)	CE (eV)	linear expression	*r*	LOD (nmol/g)
isoalloUCA	2.79	407.3	361.3	32	y = 0.133424 x + 0.000068	0.9998	0.012
isoUCA	3.11	407.3	343.3	32	y = 0.136398 x - 0.000182	0.9999	0.012
UCA	4.81	407.3	345.3	34	y = 0.039365 x - 0.000019	0.9998	0.096
alloUCA	5.04	407.4	359.3	35	y = 0.159070 x + 0.000532	0.9998	0.012
CA-Δ^4-3-one	5.76	403.3	123.2	30	y = 0.180769 x + 0.000269	0.9998	0.012
isoCA	6.23	407.3	289.3	33	y = 0.076214 x + 0.000195	0.9998	0.009
nor-CA	6.35	393.3	329.2	34	y = 0.106972 x + 0.000311	0.9996	0.015
7-oxo-DCA	6.77	405.3	289.2	35	y = 0.140445 x + 0.000021	0.9996	0.006
12-oxo-CDCA	7.94	447.3	405.3	27	y = 0.354773 x + 0.001695	0.9997	0.006
HCA	8.75	407.3	389.2	33	y = 0.007984 x + 0.000010	0.9992	0.180
3-oxo-UDCA	9.02	403.3	359.0	40	y = 0.117536 x + 0.000522	0.9997	0.015
MDCA	9.36	437.3	391.2	30	y = 0.345398 x + 0.001117	0.9999	0.006
3-oxo-CA	9.56	405.3	289.3	36	y = 0.249166 x - 0.000090	0.9999	0.009
isoUDCA	9.78	391.3	391.3	33	y = 0.250825 x - 0.000550	0.9997	0.009
d_5-CA	11.01	412.4	348.3	34			
CA	11.03	407.3	343.3	33	y = 0.112079 x + 0.000199	0.9998	0.009
alloCA	11.48	407.3	361.2	30	y = 0.139344 x + 0.000256	0.9998	0.012
CA-$\Delta^{4,6}$-3-one	11.67	385.3	341.2	26	y = 0.388488 x + 0.000259	0.9997	0.012
UDCA	11.80	437.3	391.2	30	y = 0.370329 x + 0.002153	0.9997	0.006
7-oxo-Δ^5-3β-ol	11.83	387.2	387.2	32	y = 0.222645 x + 0.000251	0.9996	0.330
12-oxo-isoLCA	12.19	391.3	373.1	-11	y = 2.15302 x + 0.006935	0.9999	0.003
HDCA	12.85	437.3	391.1	35	y = 0.551712 x + 0.002339	0.9999	0.018
12β-OH-isoLCA	13.36	391.4	345.2	36	y = 0.211340 x + 0.000752	0.9999	0.012
isoalloDCA	13.42	391.6	345.2	40	y = 0.118206 x - 0.0001058	0.9996	0.012

3,12-dioxo-DCA	13.84	389.3	371.1	-14	y = 0.916459 x + 0.003008	0.9999	0.018
DCA-Δ^4-3-one	13.88	387.3	123.2	37	y = 0.890582 x + 0.001195	0.9999	0.003
CDCA-Δ^4-3-one	14.22	387.3	369.3	28	y = 0.106055 x - 0.000380	0.9996	0.009
12β-OH-LCA	15.31	391.4	345.2	35	y = 0.655488 x + 0.002512	0.9997	0.006
isoDCA	15.33	391.2	345.3	30	y = 0.748451 x + 0.002801	0.9997	0.006
isoCDCA	15.70	437.3	391.2	30	y = 0.319910 x + 0.000798	0.9999	0.027
12-oxo-LCA	17.24	391.2	373.1	-11	y = 1.75490 x - 0.015476	0.9987	0.042
7-oxo-LCA	19.73	435.3	389.2	20	y = 0.504668 x + 0.001372	0.9998	0.012
3-oxo-alloCDCA	20.73	389.3	327.4	46	y = 0.150838 x + 0.000533	0.9999	0.084
3-oxo-alloDCA	20.74	389.4	343.3	31	y = 0.416706 x + 0.000381	0.9997	0.018
CDCA	24.34	437.3	391.2	33	y = 0.404525 x + 0.002806	0.9999	0.009
3-oxo-DCA	24.71	389.3	343.2	34	y = 0.238870 x - 0.000514	0.9998	0.066
3-oxo-CDCA	25.19	389.3	389.3	37	y = 0.508835 x + 0.000356	0.9997	0.030
alloCDCA	25.23	437.3	391.3	21	y = 0.224449 x + 0.000751	0.9997	0.012
d_5-DCA	25.55	396.4	350.3	36			
DCA	25.57	391.3	345.3	34	y = 0.244963 x + 0.000464	0.9999	0.012
alloDCA	25.98	391.3	345.3	33	y = 0.259484 x + 0.000391	0.9999	0.006
CDCA-$\Delta^{4,6}$-3-one	28.89	369.3	325.3	29	y = 0.172740 x + 0.000774	0.9999	0.006
LCA-Δ^4-3-one	30.23	371.3	123.1	32	y = 0.019466 x + 0.000039	0.9999	0.075
isoalloLCA	30.92	375.3	375.3	30	y = 1.55412 x - 0.000312	0.9998	0.030
isoLCA	30.92	375.3	375.3	30	y = 1.55412 x - 0.000312	0.9998	0.030
d_4-LCA	32.42	379.3	379.3	36			
LCA	32.70	375.3	375.3	36	y = 0.808470 x + 0.007081	0.9999	0.006
3-oxo-alloLCA	33.34	373.3	373.3	20	y = 3.91836 x + 0.002474	0.9999	0.015
3-oxo-LCA	33.34	373.4	373.4	34	y = 4.04908 x + 0.003678	0.9999	0.009
alloLCA	33.43	375.3	357.3	19	y = 0.001131 x + 0.000052	0.9975	6.3

RT, retention time; CE, collision energy; LOD, limit of detection.

Supplemental table S3. LC/MS parameters for the FA quantitation in this study

Fatty acid	RT (min)	Precursor ion (m/z)	Product ion (m/z)	CE (eV)	linear expression	*r*	LOD (nmol/g)
Pam	4.87	255.3	237.4	22	y = 0.096422 x + 0.00550	0.9995	35
Ste	6.54	283.3	265.2	23	y = 0.570706 x + 0.02964	0.9997	12
Ole	5.25	281.3	263.3	22	y = 0.486907 x + 0.01633	0.9997	8
Lin	4.34	279.3	261.3	17	y = 0.295962 x + 0.00014	0.9998	18
d_3-Pam	4.86	258.3	240.3	20			
$^{13}C_{18}$-Ste	6.51	301.3	283.4	24			

RT, retention time; CE, collision energy; LOD, limit of detection.

Supplemental table S4. Effect of method A and method B on bile acids

Bile acids (500pmol/ml)	Method A (0.2N NaOH, 40ºC 2h)		Mehtod B (1N KOH/MeOH, reflux 1h)	
	Amount collected (%)	Product Bile acid (%)	Amount collected (%)	Product Bile acid (%)
3-oxo-CA	97.8		23.2	
3-oxo-CDCA	96.3		19.5	
3-oxo-UDCA	99.7		19.8	
3-oxo-DCA	101.3		35.7	
3-oxo-LCA	95.8		50.9	
3-oxo-alloCDCA	102.6		46.8	
3-oxo-alloDCA	101.6		42.4	
3-oxo-alloLCA	98.1		28.8	
12-oxo-CDCA	82.1		0.0	
7-oxo-Δ^5-3β-ol	95.5		0.0	
CA-Δ^4-3-one	15.0	CA-$\Delta^{4,6}$-3-one 66.9	0.0	CA-$\Delta^{4,6}$-3-one 84.5
CDCA-Δ^4-3-one	29.5	CDCA-$\Delta^{4,6}$-3-one 62.8	0.0	CDCA-$\Delta^{4,6}$-3-one 86.3
DCA-Δ^4-3-one	60.2		0.0	
LCA-Δ^4-3-one	89.3		1.0	
GCA-Δ^4-3-one	70.4		0.2	
TCA-Δ^4-3-one	71.2		0.0	
GCDCA-Δ^4-3-one	82.3		0.0	
TCDCA-Δ^4-3-one	36.6		0.0	

Bile acids that changed by more than 5% before and after the treatments are shown. If the side reactant was a bile acid, the bile acid was noted and the amount after the reaction was shown.

Supplemental table S5. Free BAs produced from FA-BA ester standards after method A and method B

FA-BA (1000 pmol)	Method A				Method B			
	DCA	isoDCA	LCA	isoLCA	DCA	isoDCA	LCA	isoLCA
	(pmol found)				(pmol found)			
Ac-LCA	0	0	40	0	0	0	787	200
Ste-LCA	0	0	0	0	0	0	651	301
Ste-DCA	0	0	0	0	967	0	0	0
Ac-isoLCA	0	0	0	96	0	0	0	958
Pam-isoLCA	0	0	0	0	0	0	0	1052
Ste-isoLCA	0	0	0	0	0	0	0	984
Ole-isoLCA	0	0	0	0	0	0	0	978
Lin-isoLCA	0	0	0	0	0	0	0	1066
Ac-isoDCA	0	248	0	0	0	1061	0	0
Pam-isoDCA	0	0	0	0	0	973	0	0
Ste-isoDCA	0	0	0	0	0	964	0	0
Ole-isoDCA	0	0	0	0	0	978	0	0
Lin-isoDCA	0	0	0	0	0	989	0	0
