# Supplementary material for: Characterization of long-chain fatty acid-linked bile acids: a major conjugation form of 3β-hydroxy bile acids in feces
Source: J Lipid Res. 2022 Sep 9;63(10):100275. doi: 10.1016/j.jlr.2022.100275 (PMC9587409; doi:10.1016/j.jlr.2022.100275)
Supplement: Supplemental Figure [file mmc4.pdf]

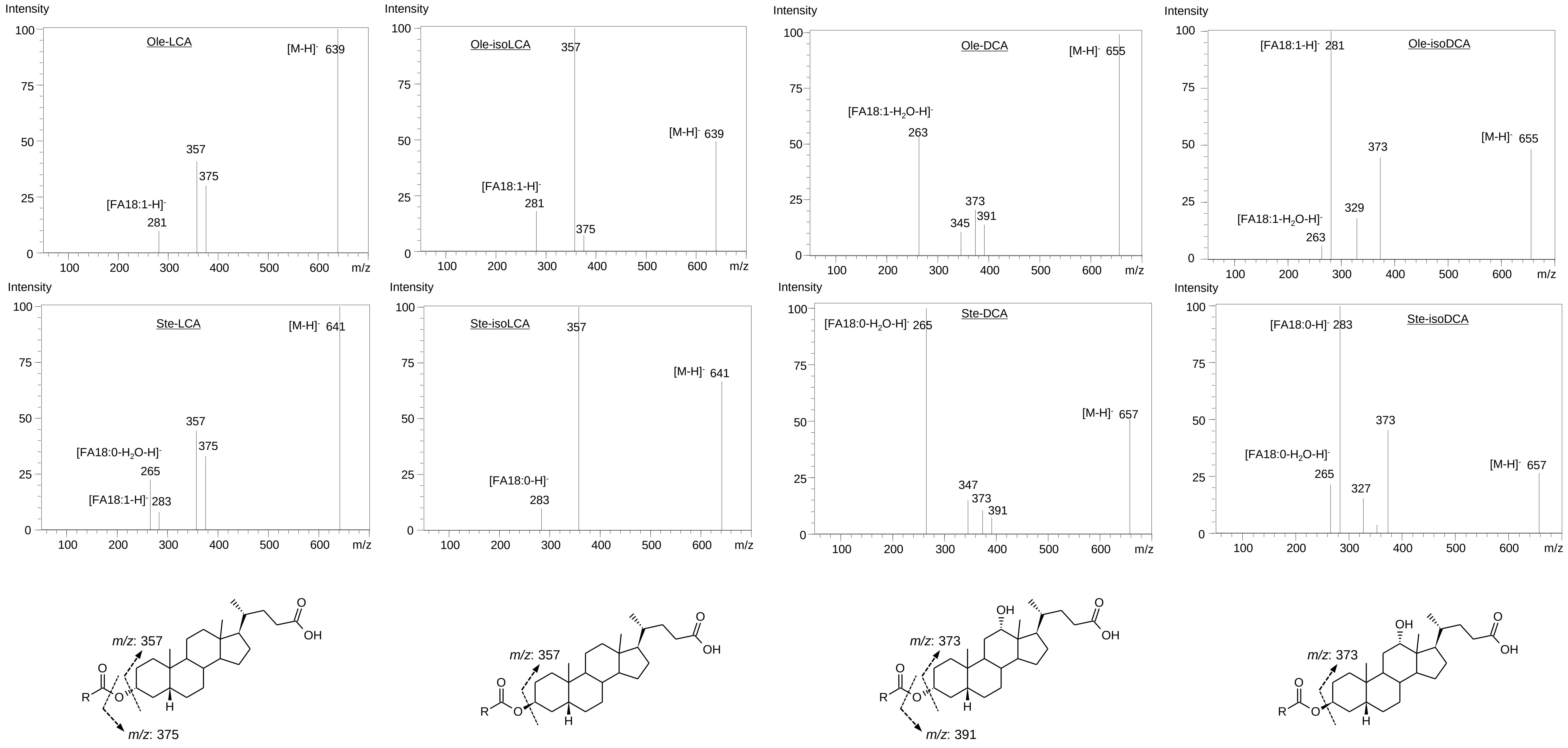


Supplemental figure S1. Negative-ion ESI MS/MS spectra of Ole-LCA, Ste-LCA, Ole-isoLCA, Ste-isoLCA, Ole-DCA, Ste-DCA, Ole-isoDCA, and Ste-isoDCA.
